# Chemoselective Lipase-Catalyzed Synthesis of Amido Derivatives from 5-Hydroxymethylfurfurylamine

**DOI:** 10.1021/acssuschemeng.3c00775

**Published:** 2023-07-06

**Authors:** Antía Pintor, Iván Lavandera, Alexey Volkov, Vicente Gotor-Fernández

**Affiliations:** † Organic and Inorganic Chemistry Department, University of Oviedo, Avenida Julián Clavería 8, Oviedo 33006, Spain; ‡ EnginZyme AB, Tomtebodavägen 6, 171 65 Solna, Sweden

**Keywords:** acylation, chemoselective process, enzyme immobilization, 5-hydroxymethylfurfurylamine, lipases

## Abstract

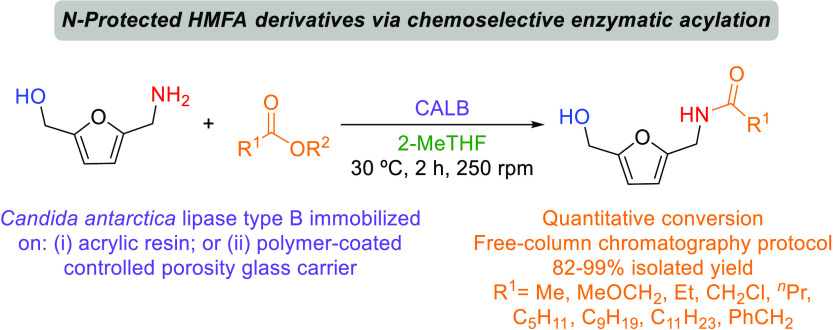

The acylations of furfurylamine and 5-hydroxymethylfurfurylamine (HMFA) have been studied finding immobilized *Candida antarctica* lipase B (CALB) as an ideal biocatalyst. CALB was used immobilized on two different supports (Novozyme 435 and EziG-CALB), with the polymer-coated controlled porosity glass carrier material from EnginZyme being an excellent carrier to yield an active and stable enzymatic preparation for the acylation of the primary amine group. The amount of the acyl donor in the reaction was a key factor to achieve the mono- and chemoselective N-protection of HMFA with large excess of ethyl acetate leading to the formation of the N,O-diacetylated product. Thus, a series of 16 nonactivated esters were used to selectively modify the amine group of HMFA, obtaining 9 hydroxy amides under mild reaction conditions and with quantitative yields through chromatography-free transformations. The influence of substrate concentration was studied, resulting in complete conversions in all cases after 22 h (100–1000 mM). Excellent results were observed at 100 and 200 mM of HMFA, while higher concentrations led to longer reaction times and, to some extent, the formation of the diacetylated product (up to 7% after 22 h at 1 M). After this optimization, a metric analysis was performed to confirm the high sustainability of the presented process (*E*-factor of 1.1 excluding solvents) upon intensification of the biotransformation to 1 g at 200 mM HMFA concentration. The possibility of obtaining orthogonally protected HMFA-derived amido esters has been achieved through a clean and sequential one-pot process using EziG-CALB, which involved the use of ethyl methoxy acetate as the nonactivated ester for N-acylation and the activated vinyl acetate for O-protection.

## Abstract 2

A series of nine hydroxy amides derived from biobased HMFA were enzymatically obtained with complete conversion and selectivity under mild conditions.

## Introduction

**Figure 1:**
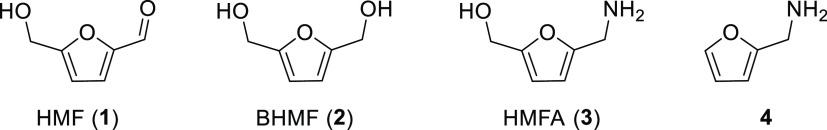
HMF (**1**), BHMF (**2**), HMFA (**3**), and furfurylamine (**4**) chemical structures.

The search for biobased chemicals and fuels from raw biomass is currently highly appealing to replace traditional fossil sources. Lignocellulosic biomass is a valuable source of platform molecules such as furan derivatives with multiple applications in chemical industry, for instance, in the manufacturing of adhesives and polymers.^1,2^ In this context, 5-hydroxymethylfurfural (HMF, **1**, Figure 1) is considered a key molecule for biomass valorization and also a versatile synthetic building block,^3−5^ with primary hydroxy and formyl groups being modular functionalities to produce different families of valuable compounds.^6,7^ Thus, chemical oxidations of HMF provide access to 2,5-diformylfuran (DFF), 5-hydroxymethyl-2-furancarboxylic acid (HMFCA), 5-formyl-2-furancarboxylic acid (FFCA), and 2,5-furandicarboxylic acid (FDCA); HMF reduction leads to 2,5-bis(hydroxymethyl)furan (BHMF, **2**); while 5-hydroxymethyl-2-furfurylamine (HMFA, **3**) can be obtained through direct HMF amination.^8−11^

Nowadays, the use of enzymes is particularly attractive in organic synthesis due to their chemo-, regio-, and stereoselective reactivity under mild reaction conditions. Particularly, straightforward and selective transformations to produce HMF derivatives have been extensively studied in the last decade.^12−18^ Thus, a vast number of biocatalysts have been identified for the production of various HMF derivatives such as oxidases,^19^ alcohol dehydrogenases,^20^ amine transaminases,^21−23^ and reductive aminases.^24^

**Scheme 1:**
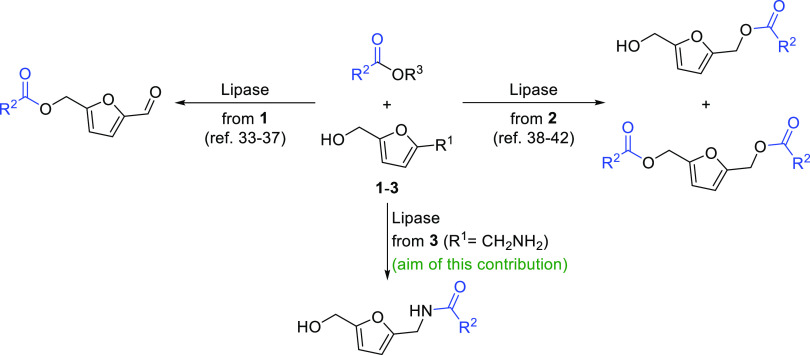
Lipase-Catalyzed Transformations Using HMF (**1**), BHMF (**2**), and HMFA (**3**)

N-Substituted furfuryl amines are of high interest as precursors to biologically active compounds,^24−26^ their chemical preparation providing excellent results via reductive amination to obtain the corresponding N-alkylated derivatives,^27−29^ while enzymatic approaches are still in their infancy for this purpose. Therefore, developing sustainable and selective synthetic routes toward N-protected furfuryl amines under mild reaction conditions would be of great interest, envisaging an acyl group as an excellent choice as it can be detached later if required. Lipases are versatile enzymes able to catalyze hydrolytic and synthetic transformations, currently finding important applications in the industrial sector.^30−32^ In recent years, the use of lipases for the synthesis of HMF derivatives has been considerably exploited by means of lipase-catalyzed (trans)esterification reactions (Scheme 1).^33−42^ The reaction between HMF and different esters or carboxylic acids allows the selective functionalization of the hydroxyl group maintaining unaltered the aldehyde functionality (Scheme 1, left).^33−37^ The (trans)esterification of BHMF, however, usually proceeds toward the formation of the corresponding diesters,^38−42^ although depending on the reaction conditions, monoesters can be selectively obtained to a certain extent (Scheme 1, right). Unfortunately, the work with the corresponding amino alcohol derivative (HMFA) remains unexplored, while the presence of the amine and hydroxyl groups offers a variety of synthetic possibilities to produce a wide range of N- or/and O-protected compounds such as, e.g., the (hydroxy) amides (Scheme 1, bottom). Based on the excellent activity and selectivity displayed by lipases, mainly *Candida antarctica* lipase type B (CALB),^43,44^ toward amide formation under mild conditions, herein, the chemoselective lipase-catalyzed acylation of the HMFA primary amine group was thoroughly investigated.

## Experimental Section

### Materials and Equipment

Chemical reagents were purchased from Sigma-Aldrich, VWR International, and Thermo Fisher Scientific and used as received. Particularly, furfurylamine and HMFA that were used as substrates for lipase-catalyzed reactions were acquired from Sigma-Aldrich. Regarding the enzyme availability, *C. antarctica* type B lipase (CALB) was used as two different immobilized forms: Novozyme 435 is supported on the resin Lewatit VP OC 1600 and it was kindly donated by Novozymes,^45^ while EziG-CALB is produced by EnginZyme and is supported on a polymer-coated controlled porosity glass carrier EziG Amber.^46^ Regarding other enzymes employed in this contribution, immobilized *Candida rugosa* lipase (CRL), immobilized *Pseudomonas cepacia* (PSL), and lyophilized lipase AK from *Pseudomonas fluorescens* (AK) were purchased from Sigma-Aldrich; *C. antarctica* type A lipase (CALA) and *Thermomyces lanuginosus* lipase (TLL) were obtained from Immozymes and Meito Sangyo, respectively, both used as immobilized preparations; finally, immobilized *Aspergillus niger* lipase (ANL) was obtained from Biocatalysts Ltd. Thin-layer chromatography (TLC) analyses were conducted using Merck Silica Gel 60 F254 precoated plates and visualized with a UV lamp and potassium permanganate or vanillin stains. Column chromatography purifications, when required, were performed using silica gel 60 (230–240 mesh).

^1^H-, ^13^C-, and DEPT NMR experiments were recorded on a Bruker AV300 MHz spectrometer using CDCl_3_ and MeOD as the solvents. All chemical shifts (δ) are given in parts per million (ppm) and referenced to the residual solvent signal as internal standard. IR spectra were recorded on a Jasco FT/IR-4700 spectrophotometer, and ν_max_ values are given in cm^–1^ for the main absorption bands of the synthesized compounds. High-resolution mass spectra (HRMS) experiments were carried out by electrospray ionization in positive mode (ESI^+^) using a Micro Tof Q spectrometer.

Gas chromatography (GC) analyses were performed on an Agilent HP6890 GC chromatograph equipped with an FID detector. A HP-1 column (30 m × 0.32 mm × 0.25 μm) was used for the determination of conversion values and product percentages (see additional information in Section 5 of the Supporting Information).

### Lipase-Catalyzed Acetylation of Furfurylamine (**4**) Using EtOAc (**5a**) in an Organic Solvent

Amine **4** (20 mg, 0.2 mmol, 100 mM) was dissolved in a hydrophobic organic solvent (2 mL) such as *tert*-butyl methyl ether (MTBE), diethyl ether (Et_2_O), ethyl acetate (EtOAc), or 2-methyltetrahydrofuran (2-MeTHF) inside an Erlenmeyer flask. Then, Novozyme 435 or EziG-CALB (20 mg, 1:1 w/w enzyme:**4** ratio) and EtOAc (59 μL, 0.6 mmol, 3 equiv) were successively added (the acyl donor was added only for the reactions with MTBE, Et_2_O, and 2-MeTHF). The reaction was shaken at 250 rpm for 2 h at 30 °C, and after this time an aliquot was taken and analyzed by GC, observing the quantitative conversion. The reaction was filtered, and the enzyme was washed with CH_2_Cl_2_ (2 × 1 mL). The filtrate was evaporated under reduced pressure, affording *N*-(furan-2-ylmethyl)acetamide (**6**) as a yellow oil. The spectroscopic data match with the ones obtained via chemical acetylation of **4** using acetic anhydride and triethylamine (see the SI). *R*_f_ (EtOAc): 0.48. IR (neat): 3274, 3077, 1646, 1544, 734, and 599 cm^–1^. ^1^H-NMR (300 MHz, CDCl_3_) δ 7.35 (dd, *J* = 2.0, 0.9 Hz, 1H), 6.31 (dd, *J* = 3.2, 1.9 Hz, 1H), 6.22 (dd, *J* = 3.2, 0.9 Hz, 1H), 5.88 (br s, 1H), 4.42 (d, *J* = 5.5 Hz, 2H), and 2.00 (s, 3H) ppm. ^13^C-NMR (75 MHz, CDCl_3_) δ 169.9 (C), 151.4 (C), 142.3 (CH), 110.6 (CH), 107.6 (CH), 36.7 (CH_2_), and 23.3 (CH_3_) ppm. ESI-TOF-sHRMS: [M + Na]^+^ calculated for C_7_H_9_NNaO_2_: 162.0532; found: 162.0525.

### General Procedure for the EziG-CALB-Catalyzed Selective N-Acylation of HMFA (**3**)

The corresponding acyl donor **5a**–**p** (0.16 mmol, 1.3 equiv) was added to a mixture of HMFA (**3**, 15 mg, 0.12 mmol, 100 mM), EziG-CALB (1:1 w/w enzyme:**3**), and 2-MeTHF (1.2 mL). The mixture was shaken for 2 h at 250 rpm at 30 °C, and after this time, the reaction crude was analyzed by TLC and GC analyses. The reaction was filtered, and the enzyme was washed with CH_2_Cl_2_ (2 × 1 mL). The filtrate was concentrated under reduced pressure, affording the corresponding hydroxy amides **7a**–**i** with excellent purities that were then fully characterized. The only exceptions were the reactions with ethyl phenylacetate (**5e**), where the reaction crude was dried on a freeze-dryer overnight and those using benzyl acetate (**5j**), 4-nitrophenyl acetate (**5k**), ethyl caprate (**5o**), and methyl laurate (**5p**) because the product formation was observed in complete conversion, but the unreacted acyl donor was not separated. For instance, the wash of the reaction crudes containing the hydroxy amides **7h** and **7i** with cold Et_2_O (3 × 2 mL) allowed the isolation of the product with excellent purity.

#### *N*-[(5-(Hydroxymethyl)furan-2-yl)methyl]acetamide (**7a**)

Yellow oil (19.8 mg, 99% isolated yield). *R*_f_ (EtOAc): 0.25. IR (neat): 3390, 3280, 1623, 1545, 998, and 796 cm^–1^. ^1^H-NMR (300 MHz, CDCl_3_) δ 6.44 (br s, 1H), 6.16 (d, *J* = 3.1 Hz, 1H), 6.12 (d, *J* = 3.2 Hz, 1H), 4.51 (s, 2H), 4.33 (d, *J* = 5.5 Hz, 2H), 3.15 (br s, 1H), and 1.94 (s, 3H) ppm. ^13^C-NMR (75 MHz, CDCl_3_) δ 170.4 (C), 154.0 (C), 151.3 (C), 108.7 (CH), 108.4 (CH), 57.3 (CH_2_), 36.8 (CH_2_), and 23.2 (CH_3_) ppm. ESI-TOF-HRMS: [M + Na]^+^ calculated for C_8_H_11_NNaO_3_: 192.0631; found: 192.0634.

#### *N*-[(5-(Hydroxymethyl)furan-2-yl)methyl]-2-methoxyacetamide (**7b**)

Yellow oil (23.3 mg, 99% isolated yield). *R*_f_ (EtOAc): 0.25. IR (neat): 3298, 2918, 2849, 1649, 1195, and 1116 cm^–1^. ^1^H-NMR (300 MHz, CDCl_3_) δ 6.93 (br s, 1H), 6.18 (d, *J* = 3.1 Hz, 1H), 6.15 (d, *J* = 3.2 Hz, 1H), 4.52 (s, 2H), 4.41 (d, *J* = 5.9 Hz, 2H), 3.88 (s, 2H), 3.37 (s, 3H), and 2.97 (br s, 1H) ppm. ^13^C-NMR (75 MHz, CDCl_3_) δ 169.8 (C), 154.2 (C), 150.9 (C), 108.6 (CH), 108.4 (CH), 71.9 (CH_2_), 59.3 (CH_3_), 57.3 (CH_2_), and 35.9 (CH_2_) ppm. ESI-TOF-HRMS: [M + Na]^+^ calculated for C_9_H_13_NNaO_4_: 222.0737; found: 222.0739.

#### *N*-[(5-(Hydroxymethyl)furan-2-yl)methyl]propionamide (**7c**)

Yellow oil (21.4 mg, 99% isolated yield). *R*_f_ (EtOAc): 0.40. IR (neat): 3350, 3173, 1645, 1539, 997, and 790 cm^–1^. ^1^H-NMR (300 MHz, CDCl_3_) δ 6.17 (d, *J* = 3.2 Hz, 1H), 6.13 (d, *J* = 3.2 Hz, 1H), 4.52 (s, 2H), 4.35 (d, *J* = 5.5 Hz, 2H), 2.95 (br s, 2H), 2.20 (q, *J* = 7.6 Hz, 2H), and 1.12 (t, *J* = 7.6 Hz, 3H) ppm. ^13^C-NMR (75 MHz, CDCl_3_) δ 174.1 (C), 154.0 (C), 151.5 (C), 108.7 (CH), 108.2 (CH), 57.3 (CH_2_), 36.7 (CH_2_), 29.6 (CH_2_), and 9.8 (CH_3_) ppm. ESI-TOF-HRMS: [M + Na]^+^ calculated for C_9_H_13_NNaO_3_: 206.0788; found: 206.0790.

#### 2-Chloro-*N*-[(5-(hydroxymethyl)furan-2-yl)methyl]acetamide (**7d**)

Yellow oil (23.7 mg, 99% isolated yield). *R*_f_ (EtOAc): 0.56. IR (neat): 3278, 3079, 1652, 1537, 1010, and 792 cm^–1^. ^1^H-NMR (300 MHz, CDCl_3_) δ 7.05 (br s, 1H), 6.19 (apparent q, *J* = 3.4 Hz, 2H), 4.54 (s, 2H), 4.43 (d, *J* = 5.7 Hz, 2H), 4.04 (s, 2H), and 3.59 (s, 1H) ppm. ^13^C-NMR (75 MHz, CDCl_3_) δ 166.2 (C), 154.2 (C), 150.4 (C), 108.9 (CH), 108.8 (CH), 57.5 (CH_2_), 42.6 (CH_2_), and 37.0 (CH_2_) ppm. ESI-TOF-HRMS: [M + Na]^+^ calculated for C_8_H_10_ClNNaO_3_: 226.0241; found: 226.0245.

#### *N*-[(5-(Hydroxymethyl)furan-2-yl)methyl]-2-phenylacetamide (**7e**)

White powder (28.6 mg, 99% isolated yield). *R*_f_ (EtOAc): 0.65. Mp: decomposition observed between 141 and 161 °C. IR (neat): 3355, 3280, 1631, 1539, 1003, 999, and 691 cm^–1^. ^1^H-NMR (300 MHz, CDCl_3_) δ 7.40–7.19 (m, 5H), 6.16 (d, *J* = 3.2 Hz, 1H), 6.07 (d, *J* = 3.1 Hz, 1H), 5.98 (br s, 1H), 4.51 (s, 2H), 4.35 (d, *J* = 5.7 Hz, 2H), and 3.57 (s, 2H). ^13^C-NMR (75 MHz, CDCl_3_) δ 171.2 (C), 153.9 (C), 151.3 (C), 134.7 (C), 129.6 (2CH), 129.1 (2CH), 127.5 (CH), 108.7 (CH), 108.2 (CH), 57.4 (CH_2_), 43.7 (CH_2_), and 36.9 (CH_2_) ppm. ESI-TOF-HRMS: [M + Na]^+^ calculated for C_14_H_15_NNaO_3_: 268.0944; found: 268.0947.

#### *N*-[(5-(Hydroxymethyl)furan-2-yl)methyl]butyramide (**7f**)

Brown powder (20.8 mg, 90% isolated yield). *R*_f_ (EtOAc): 0.50. Mp: 79–81 °C. IR (neat): 3277, 2962, 2931, 2872, 1625, 1543, 1275, 996, and 758 cm^–1^. ^1^H-NMR (300 MHz, MeOD-*d*_4_) δ 6.22 (d, *J* = 3.2 Hz, 1H), 6.17 (d, *J* = 3.2 Hz, 1H), 4.46 (s, 2H), 4.33 (s, 2H), 2.18 (t, *J* = 7.4 Hz, 2H), 1.57–1.70 (sept, *J* = 7.4 Hz, 2H), 0.93 (t, *J* = 7.4 Hz, 3H). ^13^C-NMR (75 MHz, MeOD-*d*_4_) δ 174.5 (C), 154.1 (C), 151.5 (C), 107.8 (CH), 107.4 (CH), 56.0 (CH_2_), 37.4 (CH_2_), 35.8 (CH_2_), 18.9 (CH_2_), 12.6 (CH_3_). ESI-TOF-HRMS: [M + Na]^+^ calculated for C_10_H_15_NNaO_3_: 220.0944; found: 220.0939.

#### *N*-[(5-(Hydroxymethyl)furan-2-yl)methyl]hexanamide (**7g**)

Orange powder (21.7 mg, 82% isolated yield). *R*_f_ (EtOAc): 0.63. Mp: 94–97 °C. IR (neat): 3283, 2954, 2930, 2871, 2449, 1623, 1540, and 1022 cm^–1^. ^1^H-NMR (300 MHz, MeOD-*d*_4_) δ 6.22 (d, *J* = 3.2 Hz, 1H), 6.17 (d, *J* = 3.1 Hz, 1H), 4.46 (s, 2H), 4.33 (s, 2H), 2.20 (t, *J* = 7.5 Hz, 2H), 1.61 (quint, *J* = 7.5 Hz, 2H), 1.39–1.21 (m, 4H), 0.91 (t, *J* = 6.8 Hz, 3H). ^13^C-NMR (75 MHz, MeOD-*d*_4_) δ 174.7 (C), 154.1 (C), 151.5 (C), 107.8 (CH), 107.4 (CH), 56.0 (CH_2_), 35.8 (CH_2_), 35.5 (CH_2_), 31.1 (CH_2_), 25.3 (CH_2_), 22.0 (CH_2_), 12.9 (CH_3_). ESI-TOF-HRMS: [M + Na]^+^ calculated for C_12_H_19_NNaO_3_: 248.1257; found: 248.1258.

#### *N*-[(5-(Hydroxymethyl)furan-2-yl)methyl]decanamide (**7h**)

Yellow powder (29.8 mg, 90% isolated yield). *R*_f_ (50% EtOAc/Hexane): 0.21. Mp: 115–118 °C. IR (neat): 3283, 2918, 2849, 1626, 1542, 1002, and 808 cm^–1^. ^1^H-NMR (300 MHz, MeOD-*d*_4_) δ 6.22 (d, *J* = 3.2 Hz, 1H), 6.17 (d, *J* = 3.1 Hz, 1H), 4.46 (s, 2H), 4.33 (s, 2H), 2.20 (t, *J* = 7.5 Hz, 2H), 1.61 (quint, *J* = 6.8 Hz, 2H), 1.30 (d, *J* = 3.5 Hz, 12H), 0.90 (t, *J* = 6.8 Hz, 3H). ^13^C-NMR (75 MHz, MeOD-*d*_4_) δ 175.2 (C), 154.6 (C), 152.1 (C), 108.4 (CH), 107.9 (CH), 56.5 (CH_2_), 36.3 (CH_2_), 36.1 (CH_2_), 32.2 (CH_2_), 29.8 (CH_2_), 29.6 (CH_2_), 29.5 (CH_2_), 29.4 (CH_2_), 26.1 (CH_2_), 22.9 (CH_2_), 13.6 (CH_3_). ESI-TOF-HRMS: [M + Na]^+^ calculated for C_16_H_27_NNaO_3_: 304.1883; found: 304.1883.

#### *N*-[(5-(Hydroxymethyl)furan-2-yl)methyl]dodecanamide (**7i**)

Pale yellow powder (33.1 mg, 91% isolated yield). *R*_f_ (50% EtOAc/Hexane): 0.26. Mp: 119–121 °C. IR (neat): 3282, 2918, 2849, 1624, 1464, 1199, and 1002 cm^–1^. ^1^H-NMR (300 MHz, MeOD-*d*_4_) δ 6.22 (d, *J* = 3.2 Hz, 1H), 6.17 (d, *J* = 3.2 Hz, 1H), 4.46 (s, 2H), 4.33 (s, 2H), 2.20 (t, *J* = 7.5 Hz, 2H), 1.60 (apparent t, *J* = 7.3 Hz, 2H), 1.30 (d, *J* = 4.4 Hz, 16H), 0.90 (t, *J* = 6.8 Hz, 3H). ^13^C-NMR (75 MHz, MeOD-*d*_4_) δ 175.2 (C), 154.6 (C), 152.1 (C), 108.4 (CH), 107.9 (CH), 56.5 (CH_2_), 36.3 (CH_2_), 36.1 (CH_2_), 32.2 (CH_2_), 29.9 (2CH_2_), 29.8 (CH_2_), 29.6 (CH_2_), 29.6 (CH_2_), 29.4 (CH_2_), 26.1 (CH_2_), 22.9 (CH_2_), 13.6 (CH_3_). ESI-TOF-HRMS: [M + Na]^+^ calculated for C_18_H_31_NNaO_3_: 332.2196; found: 332.2196.

### Study of the Influence of the Substrate Concentration in the EziG-CALB-Catalyzed N-Acylation of HMFA (**3**)

Ethyl acetate (1.3 equiv) was added to a mixture of HMFA (**3**, 30–150 mg, 0.24–1.2 mmol, 200–1000 mM), EziG-CALB (15 mg), and 2-MeTHF (1.2 mL). The corresponding mixture was shaken between 2 and 22 h at 30 °C and 250 rpm, taking aliquots regularly that were analyzed by GC (see the SI). The reactions were stopped once the complete disappearance of the starting material was achieved. In all cases, hydroxy amide **7a** was obtained as a major component (93–99% yield). Higher concentrations of the substrate favored the formation of the diacetylated product **8a** to some extent, particularly under prolonged reaction times (1–7%).

### Scale-Up of the EziG-CALB-Catalyzed N-Acylation of HMFA (**3**)

Ethyl acetate (1.0 mL, 10.22 mmol, 1.3 equiv) was added to a mixture of HMFA (**3**, 1.00 g, 7.87 mmol, 200 mM), EziG-CALB (500 mg, 1:2 w/w enzyme:**3**), and 2-MeTHF (38.3 mL). The mixture was shaken for 5 h at 250 rpm at 30 °C, and after this time, the reaction crude was analyzed by GC analyses. The reaction was filtered, and the enzyme was washed with CH_2_Cl_2_ (2 × 10 mL). The filtrate was concentrated under reduced pressure, affording the hydroxy amide **7a** (1.13 g, 85% yield) and 99% purity through GC analysis.

### General Procedure for the EziG-CALB-Catalyzed Diacetylation of HMFA (**3**)

A suspension of HMFA (**3**, 15 mg, 0.12 mmol, 100 mM) and EziG-CALB (1:1 w/w enzyme:**3**) in EtOAc (1.2 mL) was shaken for 2 h at 250 rpm at 30 °C, and after this time, the enzyme was filtered and washed with CH_2_Cl_2_ (2 × 1 mL). The filtrate was concentrated under reduced pressure, affording amido ester **8a** with excellent purity that was fully characterized.

#### 5-(Acetamidomethyl)furan-2-(yl)methyl Acetate (**8a**)

Yellow oil (78 mg, 99% isolated yield). *R*_f_ (EtOAc): 0.65. IR (neat): 2988, 2940, 1733, 1370, 1230, and 1048 cm^–1^. ^1^H-NMR (300 MHz, CDCl_3_) δ 6.33 (d, *J* = 3.2 Hz, 1H), 6.19 (d, *J* = 3.1 Hz, 1H), 5.93 (br s, 1H), 4.99 (s, 2H), 4.40 (d, *J* = 5.5 Hz, 2H), 2.07 (s, 3H), and 2.00 (s, 3H) ppm. ^13^C-NMR (75 MHz, CDCl_3_) δ 170.7 (C), 169.9 (C), 152.3 (C), 149.3 (C), 111.8 (CH), 108.6 (CH), 58.2 (CH_2_), 36.7 (CH_2_), 23.3 (CH_3_), and 21.0 (CH_3_) ppm. ESI-TOF-HRMS: [M + Na]^+^ calculated for C_10_H_13_NNaO_4_: 234.0739; found: 234.0737.

### General Procedure for the One-Pot Sequential Double Acylation of HMFA Using EziG-CALB

EziG-CALB (20 mg) and ethyl methoxy acetate (**5b**, 24 μL, 0.21 mmol, 1.3 equiv) were added to a solution of HMFA (**3**, 20 mg, 0.16 mmol, 100 mM) in 2-MeTHF (1.5 mL). The mixture was shaken for 2 h at 30 °C and 250 rpm, until the complete consumption of the starting amine was observed by GC analysis toward the formation of methoxyacetamide **7b**. Thereafter, vinyl acetate (**9**, 44 μL, 0.47 mmol, 3 equiv) was added to the reaction mixture, and the reaction was shaken for an additional 2 h at 30 °C and 250 rpm observing the disappearance of **7b** and the formation of product **10** (GC analysis). The reaction was filtered, and the enzyme washed with CH_2_Cl_2_ (2 × 1 mL). The filtrate was concentrated under reduced pressure, recovering **10** with excellent purity (36.4 mg, 96% isolated yield).

#### {5-[(2-Methoxyacetamido)methyl]furan-2-yl}methyl Acetate (**10**)

Yellow oil. *R*_f_ (EtOAc): 0.60. IR (neat): 3343, 3135, 3112, 1739, 1658, 1262, and 751 cm^–1^. ^1^H-NMR (300 MHz, CDCl_3_) δ 6.85 (br s, 1H), 6.33 (d, *J* = 3.2 Hz, 1H), 6.21 (d, *J* = 3.2 Hz, 1H), 5.00 (s, 2H), 4.46 (d, *J* = 5.8 Hz, 2H), 3.92 (s, 2H), 3.41 (s, 3H), and 2.07 (s, 3H) ppm. ^13^C-NMR (75 MHz, CDCl_3_) δ 170.7 (C), 169.6 (C), 152.0 (C), 149.3 (C), 111.7 (CH), 108.7 (CH), 72.0 (CH_2_), 59.3 (CH_3_), 58.2 (CH_2_), 35.9 (CH_2_), and 21.0 (CH_3_) ppm. ESI-TOF-HRMS: [M + Na]^+^ calculated for C_11_H_15_NNaO_5_: 264.0844; found: 264.0842.

## Results and Discussion

**Figure 2:**
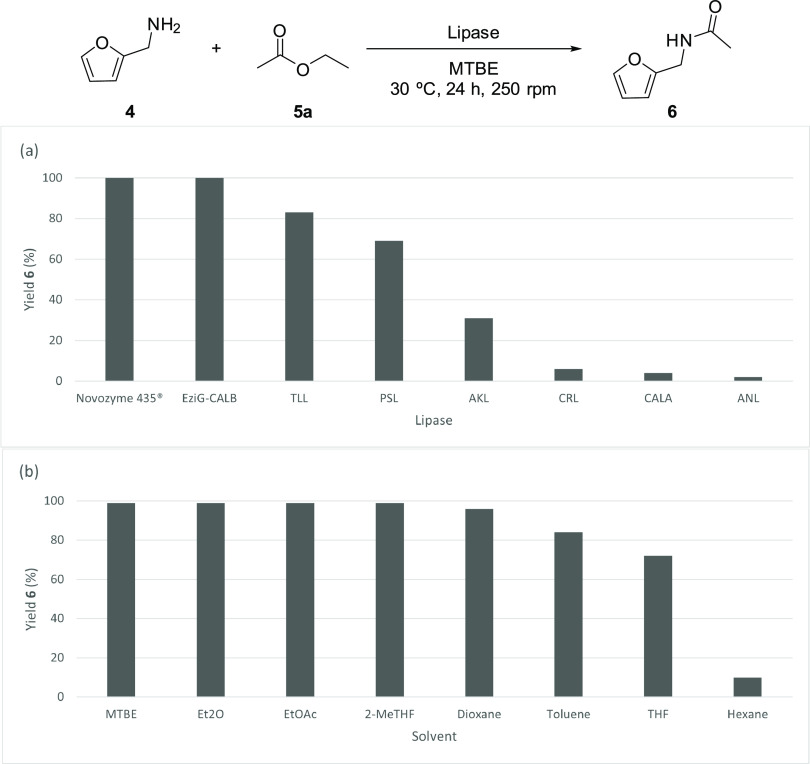
Lipase-catalyzed acetylation of **4**: (a) under standard conditions (100 mM **4** in MTBE, 3 equiv of **5a**, and lipase:**4** (1:1 w/w) at 30 °C and 250 rpm for 24 h), and (b) solvent screening using 100 mM **4**, 3 equiv of **5a**, and EziG-CALB (1:1 w/w enzyme:**4**) at 30 °C and 250 rpm for 2 h.

In a first set of experiments, furfurylamine (**4**, 100 mM) was selected as a model substrate due to its commercial availability at low price, while the presence of a single reactive group facilitated the identification of active lipases for the acylation of the primary amino group. Due to the high reactivity of amines, the use of nonactivated acylating agents such as EtOAc was recommended to avoid the background reaction.^43^ Thus, a lipase screening was performed under standard reaction conditions (3 equiv of EtOAc, MTBE, 30 °C, 24 h and 250 rpm) to produce *N*-(furan-2-ylmethyl)acetamide (**6**). The results are depicted in Figure 2a, while a more comprehensive data can be found in Table S1. CALB was identified as the most efficient enzyme under the chosen conditions, which was in line with the excellent reactivity found in the literature for the classical kinetic resolution of racemic furfuryl amines using the commercial preparation Novozyme 435 as the biocatalyst.^47−49^ The reaction with the Novozyme 435 preparation led to quantitative conversion into acetamide **6** under nonoptimized reaction conditions, motivating us to use a recently described immobilized CALB based on EnginZyme technology.^46^ Gladly, also quantitative conversion was obtained, being both superior results than the ones obtained with other lipases (TLL, PSL, AKL, CRL, CALA, and ANL).

**Figure 3:**
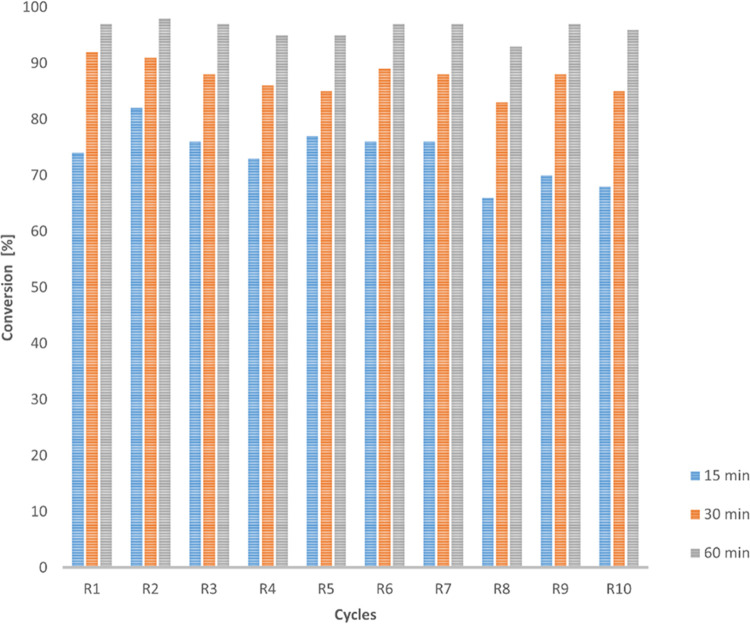
Recycling studies for the acetylation of furfurylamine (**4**, 20 mg, 100 mM) using immobilized EziG-CALB (20 mg), EtOAc (3 equiv) in 2-MeTHF at 30 °C and 250 rpm. Conversion values for the 10 reactions were determined by GC analyses of the reaction crudes, recovering the enzyme after each use by filtration and wash with 2-MeTHF.

In order to obtain further information about the synthetic benefits of both CALB preparations, a time study was performed finding that very short reaction times (30 min, Figure S1) led to conversions over 90% for both catalysts, requiring only 2 h for a quantitative conversion of the starting compound. At this point, and inside a collaborative project, we decided to explore the synthetic possibilities of EziG-CALB more in depth. Thus, the use of these immobilized biocatalysts was prioritized, and solvent screening was performed (Figure 2b and Table S2). Complete conversions were reached after 2 h with a series of ethers such as MTBE, Et_2_O, and 2-MeTHF. Remarkably, this last solvent has been previously identified as an ideal biorenewable medium for hydrolase-catalyzed reactions.^50^ Alternatively, EtOAc was also evaluated, presenting the advantage of being utilized as both the acyl donor and solvent that would simplify the reaction protocol. Exploring the potential of EziG-CALB preparation, a recyclability study was performed over 10 cycles in the acetylation of furfurylamine (Figure 3), granting over 93% conversion after 1 h of reaction in all of the experiments.

Next, the best enzymes found for the acetylation of amine **4** were applied in the lipase-catalyzed acetylation of 5-hydroxymethylfurfurylamine (**3**). Due to the existence of two competing functional groups in this molecule (alcohol *vs* amine), and in an attempt to develop a chemoselective process,^51^ a lower amount of the acyl donor, i.e., ethyl acetate, was envisaged to avoid the occurrence of the N,O-diacetylation reaction (Table 1). In fact, there are examples in the literature where the CALB-catalyzed acylation of amino alcohols has led to variable mixtures of N- and O-acylated compounds depending on the reaction medium and substrate employed.^52,53^ Gratifyingly, in our case, using 1.3 equiv of EtOAc and biobased 2-MeTHF as the solvent, both immobilized CALB preparations provided complete conversions (entries 2 and 3) toward N-acetylated product **7a**, while TLL, PSL, and lipase AK from *P. fluorescens* led to moderate to high conversion values (31–83%, entries 4–6).

**Table 1:**
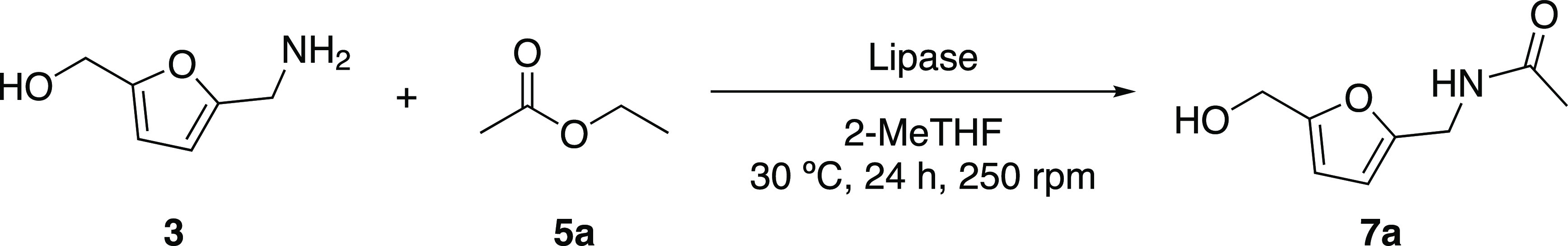
Screening of Lipases for the Enzymatic Acetylation of HMFA (**3**)^a^

entry	enzyme	**7a** (%)^b^
1		<3
2	Novozyme 435	>99
3	EziG-CALB	>99
4	TLL	83
5	PSL	69
6	AK	31

a Reaction conditions: **3** (100 mM in 2-MeTHF), 1.3 equiv of **5a**, and 1:1 w/w enzyme:**3** ratio for 24 h at 30 °C and 250 rpm.

b Conversion calculated by GC analyses of the reaction crudes (see the SI for details).

Interestingly, the reactions stopped at the chemo- and monoselective N-protection of HMFA, allowing to obtain the hydroxy amide **7a** with excellent yield. Importantly, when the reactions were carried out in EtOAc as the solvent, the formation of the amido ester **8a** was observed as the unique product, which was clearly observed due to the shift of the methylene signal, previously assigned to the free hydroxyl group at 4.51 ppm, that in the ester appeared at 4.99 ppm (see Experimental Section and NMR spectra in the SI). This fact highlights the importance of the selection of adequate reaction conditions for selective biotransformations.

**Figure 4:**
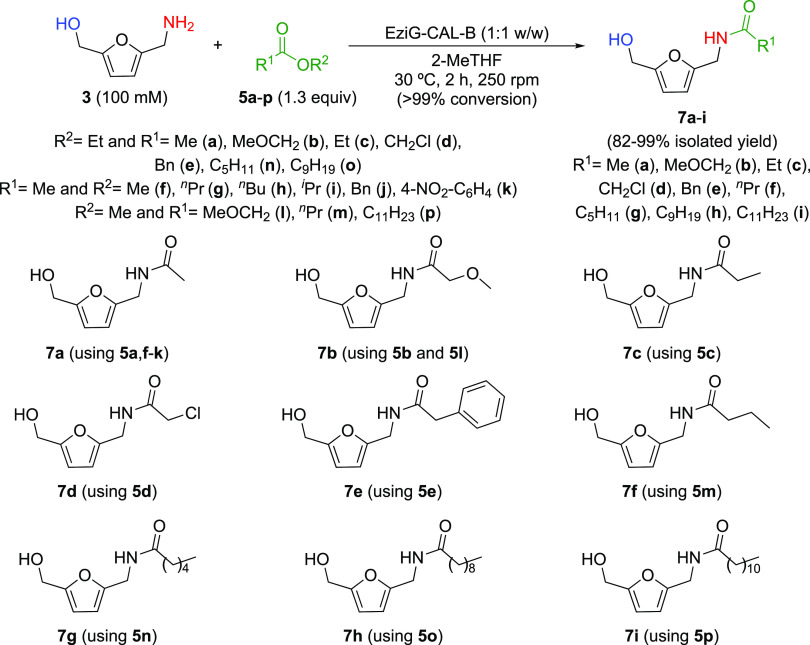
Scope of the EziG-CALB-catalyzed chemoselective acylation of HMFA.

At this point, the scope of the lipase-catalyzed acylation of HMFA was investigated with a variety of acyl donors to synthesize a family of HMFA-derived amides (**7a**–**i**) and to compare the reactivity when presenting different structural motifs in the acyl donor (Figure 4 and Table S3). All of the reactions were carried out at 100 mM substrate concentration, mild reaction conditions (30 °C), and short reaction times (2 h) using EziG-CALB (1:1 weight ratio enzyme:substrate) and 1.3 equiv of acyl donors **5a**–**p** in biorenewable 2-MeTHF. Interestingly, complete conversions were achieved in all cases for the selective N-protection of HMFA, leading to hydroxy amides **7a**–**i** with full conversion, quantitative yields, and in most of the cases products were recovered through simple enzyme filtration and subsequent solvent evaporation.

On one hand, the use of acyl donors **5a**–**e**,**m**–**p** bearing different acyl groups (Figure 4; R^1^ = Me, MeOCH_2_, Et, ClCH_2_, Ph, ^*n*^Pr, pentyl, nonyl, and undecyl) led to excellent results providing a straightforward approach to a series of HMFA-derived amides **7a**–**i** that were recovered with excellent purity after a column chromatography-free protocol. Interestingly, halogenated derivative **7d** was synthesized, that can be easily envisaged as a compound for further selective transformations. On the other hand, the use of different alkoxy groups in the nonactivated esters to produce **7a** with **5f**–**k** (R^2^ = Me, ^*n*^Pr, ^*n*^Bu, ^*i*^Pr, Bn, and 4-NO_2_C_6_H_4_), **7b** with **5l** (R^2^ = Me), **7f** with **5m** (R^2^ = Me), and **7i** with **5p** (R^2^ = Me) was found to be compatible with EziG-CALB, affording in all cases the desired hydroxy amides with excellent yields. Only a very sterically hindered ester such as *tert*-butyl acetate did not lead to any conversion toward **7a**.

Before proceeding with the scale-up of the reaction, lipase-catalyzed acetylation of HMFA was tested at different substrate concentrations (200, 300, 400, 500, and 1000 mM). The temperature, EtOAc ratio, the amount of EziG-CALB, and solvent (30 °C, 1.3 equiv, 15 mg, and 1.2 mL, respectively) were kept constant. Monitoring the reactions using GC analyses showed complete conversions in all cases, although longer reaction times (up to 22 h) were required for more concentrated transformations. Unfortunately, the use of higher HMFA concentrations and longer reaction times led to the formation of moderate amounts of the diacetylated product **8a** (up to 7%, see Figure S2). Thereafter, the lipase-catalyzed process with 200 mM HMFA was conducted at 1 g scale (7.87 mmol), obtaining the desired hydroxy amide **7a** after 5 h and a simple free-column work-up consisting of enzyme filtration and solvent evaporation (85% isolated yield).

An environmental impact analysis was performed, making use of the *E*-factor concept,^54^ for the biotransformation at 1 g scale (Figures S3 and S4). Taking into account the reagents, catalyst, and solvents employed in this protocol, we could confirm that our methodology presented a value of 53.6, with the most-contributing to the factor (97.9%) being the organic solvents that were used in the reaction medium and downstream process to wash the enzyme. In fact, excluding solvents, our enzymatic method presented an excellent value of 1.1, demonstrating its high potential, especially if the organic solvents could be reutilized.

**Scheme 2:**
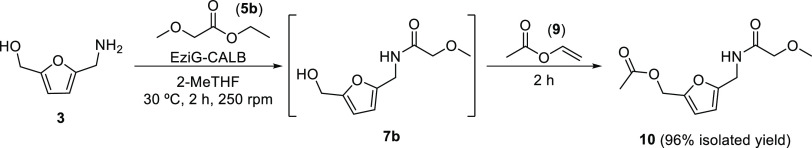
One-Pot Two-Step Enzymatic Process to Obtain Orthogonally Protected HMFA Amido Ester **10**

Last but not the least, we decided to explore the possibility of accessing the orthogonally protected amido ester using a fully enzymatic approach, ideally performed in one pot (Scheme 2). For that reason, we took advantage of the excellent chemoselectivity displayed by EziG-CALB in the monoselective N-acylation of HMFA using ethyl methoxy acetate (**5b**) in 2-MeTHF. Once the reaction reached complete conversion to hydroxy amide **7b** after 2 h, an activated acyl donor such as vinyl acetate (**9**, 3 equiv) was added. Under these conditions and after 2 h of additional reaction time, amide **10** was isolated (96%) possessing two different acyl moieties as O- and N-substitutions. The demonstrated straightforward reaction sequence to such class of compounds opens the door for easy access to, on one hand, different amido esters by selecting the proper (non) activated esters, and on the other hand, O-protected HMFA derivatives after selective N-deprotection, if required.

## Conclusions

Lipases are suitable enzymes for the functionalization of amines, which is highly attractive when valuable building blocks and pharmacologically active molecules are synthesized. This is the case for *C. antarctica* lipase type B (CALB), that is usually the enzyme of choice for amide formation, and herein it was employed to prepare a series of amides starting from furfurylamine and especially from the bifunctional 5-hydroxymethylfurfurylamine. Two immobilized preparations of CALB, one on an acrylic resin support and the other on a glass porous material carrier, have yielded chemoselective functionalization of the primary amino group of HMFA. Remarkably, EziG-CALB was shown to be an excellent biocatalyst that can be used for several cycles in a biobased organic solvent such as 2-MeTHF without significant loss of the enzyme activity. Controlling the amount of the acyl donor was found to be the key factor for selective functionalizations, and the use of only 1.3 equiv of EtOAc led to *N*-[(5-(hydroxymethyl)furan-2-yl)methyl]acetamide (**7a**). Higher acyl donor concentrations provided straightforward access to the corresponding amido ester **8a**. Remarkably, using the optimized reaction conditions, chemoselective N-acylation of HMFA was demonstrated in the synthesis of nine hydroxy amides with different (functionalized) acyl substituents. This approach was shown to be easily scalable to 1 g of substrate, and feasible at high substrate concentrations (up to 1 M). Moreover, it is characterized with a good environmental impact as demonstrated by the *E*-factor calculation of the free-column scale-up process (53.6 and 1.1, including and excluding solvents, respectively). At last, the formation of orthogonally N,O-diprotected amido ester **10** was demonstrated through a one-pot two-step transformation, via sequential addition of ethyl methoxy acetate and vinyl acetate as acyl donors for N- and O-protection, respectively, under very mild reaction conditions. This methodology can be envisaged as a promising tool for the design of selectively modified HMFA derivatives.

## Supporting Information Available

Structures of all chemical compounds studied, optimization of the reaction conditions, development of GC analytical methods, environmental assessment calculations, and copies of ^1^H-NMR, ^13^C-NMR, and DEPT NMR spectra of synthesized derivatives (PDF)

## Abbreviations

CALB: *Candida antarctica* lipase type B
equiv: equivalents
EziG: EnginZyme
HMF: 5-hydroxymethylfurfural
HMFA: 5-hydroxymethylfurfurylamine

## References

[ref1] Hou Q.; Qi X.; Zhen M.; Qian H.; Nie Y.; Bai C.; Zhang S.; Bai X.; Ju M. Biorefinery roadmap based on catalytic production and upgrading 5-hydroxymethylfurfural. Green Chem. 2021, 23, 119–231. 10.1039/D0GC02770G.

[ref2] Rosenfeld C.; Konnerth J.; Sailer-Kronlachner W.; Rosenau T.; Potthast A.; Solt P.; van Herwijnen H. W. G. Hydroxymethylfurfural and its derivatives: Potential key reactants in adhesives. ChemSusChem 2020, 13, 5408–5422. 10.1002/cssc.202001539. 32755049 PMC7693354

[ref3] Rosatella A. A.; Simeonov S. P.; Frade R. F. M.; Afonso C. A. M. 5-Hydroxymethylfurfural (HMF) as a building block platform: Biological properties, synthesis and synthetic applications. Green Chem. 2011, 13, 754–793. 10.1039/C0GC00401D.

[ref4] van Putten R.-J.; van der Waal J. C.; de Jong E.; Rasrendra C. B.; Heeres H. J.; de Vries J. G. Hydroxymethylfurfural, a versatile platform chemical made from renewable resources. Chem. Rev. 2013, 113, 1499–1597. 10.1021/cr300182k. 23394139

[ref5] Xu C.; Paone E.; Rodríguez-Padrón D.; Luque R.; Mauriello F. Recent catalytic routes for the preparation and the upgrading of biomass derived furfural and 5-hydroxymethylfurfural. Chem. Soc. Rev. 2020, 49, 4273–4306. 10.1039/D0CS00041H. 32453311

[ref6] Kong X.; Zhu Y.; Fang Z.; Kozinski J. A.; Butler I. S.; Xu L.; Song H.; Wei X. Catalytic conversion of 5-hydroxymethylfurfural to some value-added derivatives. Green Chem. 2018, 20, 3657–3682. 10.1039/C8GC00234G.

[ref7] Dutta S. Valorization of biomass-derived furfurals: Reactivity patterns, synthetic strategies, and applications. Biomass Convers. Biorefin. 2021 10.1007/s13399-021-01924-w.

[ref8] Zhang Z.; Deng K. Recent advances in the catalytic synthesis of 2,5-furandicarboxylic acid and its derivatives. ACS Catal. 2015, 5, 6529–6544. 10.1021/acscatal.5b01491.

[ref9] Totaro G.; Sisti L.; Marchese P.; Colonna M.; Romano A.; Gioia C.; Vannini M.; Celli A. Current advances in the sustainable conversion of 5-hydroxymethylfurfural into 2,5-furandicarboxylic acid. ChemSusChem 2022, 15, e202200501 10.1002/cssc.202200501. 35438242 PMC9400982

[ref10] Tran P. H. Recent approaches in the catalytic transformation of biomass-derived 5-hydroxymethylfurfural into 2,5-diformylfuran. ChemSusChem 2022, 15, e202200220 10.1002/cssc.202200220. 35307983

[ref11] Truong C. C.; Mishra D. K.; Suh Y.-W. Recent catalytic advances on the sustainable production of primary furanic amines from the one-pot reductive amination of 5-hydroxymethylfurfural. ChemSusChem 2023, 16, e202201846 10.1002/cssc.202201846. 36354122

[ref12] Domínguez de María P.; Guajardo N. Biocatalytic valorization of furans: Opportunities for inherently unstable substrates. ChemSusChem 2017, 10, 4123–4134. 10.1002/cssc.201701583. 28869788

[ref13] Hu L.; He A.; Liu X.; Xia J.; Xu J.; Zhou S.; Xu J. Biocatalytic transformation of 5-hydroxymethylfurfural into high-value derivatives: Recent advances and future aspects. ACS Sustainable Chem. Eng. 2018, 6, 15915–15935. 10.1021/acssuschemeng.8b04356.

[ref14] Troiano D.; Orsat V.; Dumont M.-J. Status of biocatalysis in the production of 2,5-furandicarboxylic acid. ACS Catal. 2020, 10, 9145–9169. 10.1021/acscatal.0c02378.

[ref15] Zhou Y.; Wu S.; Bornscheuer U. T. Recent advances in (chemo)enzymatic cascades for upgrading bio-based resources. Chem. Commun. 2021, 57, 10661–10674. 10.1039/D1CC04243B. 34585190

[ref16] Cunha J. T.; Romaní A.; Domingues L. Whole Cell Biocatalysis of 5-Hydroxymethylfurfural for Sustainable Biorefineries. Catalysts 2022, 12, 202 10.3390/catal12020202.

[ref17] Li N.; Zong M.-H. (Chemo)biocatalytic upgrading of biobased furanic platforms to chemicals, fuels, and materials: A comprehensive review. ACS Catal. 2022, 12, 10080–10114. 10.1021/acscatal.2c02912.

[ref18] Saikia K.; Rathankumar A. K.; Kumar P. S.; Varjani S.; Nizar M.; Lenin R.; George J.; Vaidyanathan V. K. Recent advances in biotransformation of 5-hydroxymethylfurfural: Challenges and future aspects. J. Chem. Technol. Biotechnol. 2022, 97, 409–419. 10.1002/jctb.6670.

[ref19] Qin Y.-Z.; Li Y.-M.; Zong M.-H.; Wu H.; Li N. Enzyme-catalyzed selective oxidation of 5-hydroxymethylfurfural (HMF) and separation of HMF and 2,5-diformylfuran using deep eutectic solvents. Green Chem. 2015, 17, 3718–3722. 10.1039/C5GC00788G.

[ref20] Chen D.; Cang R.; Zhang Z.-D.; Huang H.; Zhang Z.-G.; Ji X.-J. Efficient reduction of 5-hydroxymethylfurfural to 2,5-bis (hydroxymethyl) furan by a fungal whole-cell biocatalyst. Mol. Catal. 2021, 500, 111341 10.1016/j.mcat.2020.111341.

[ref21] Dunbabin A.; Subrizi F.; Ward J. M.; Sheppard T. D.; Hailes H. C. Furfurylamines from biomass: transaminase catalysed upgrading of furfurals. Green Chem. 2017, 19, 397–404. 10.1039/C6GC02241C.

[ref22] Petri A.; Masia G.; Piccolo O. Biocatalytic conversion of 5-hydroxymethylfurfural: Synthesis of 2,5-bis(hydroxymethyl)furan and 5-(hydroxymethyl)furfurylamine. Catal. Commun. 2018, 114, 15–18. 10.1016/j.catcom.2018.05.011.

[ref23] Wang Z.; Chai H.; Ren J.; Tao Y.; Li Q.; Ma C.; Ai Y.; He Y. Biocatalytic valorization of biobased 5-hydroxymethylfurfural to 5-hydroxymethyl-2-furfurylamine in a three-constituent deep eutectic solvent–water system. ACS Sustainable Chem. Eng. 2022, 10, 8452–8463. 10.1021/acssuschemeng.2c01481.

[ref24] Yang Z.-Y.; Hao Y.-C.; Hu S.-Q.; Zong M.-H.; Chen Q.; Li N. Direct reductive amination of biobased furans to *N*-substituted furfurylamines by engineered reductive aminase. Adv. Synth. Catal. 2021, 363, 1033–1037. 10.1002/adsc.202001495.

[ref25] Feriani A.; Gaviraghi G.; Toson G.; Mor M.; Barbieri A.; Grana E.; Boselli C.; Guarneri M.; Simoni D.; Manfredini S. Cholinergic agents structurally related to furtrethonium. 2. Synthesis and antimuscarinic activity of a series of *N*-[5-[(1′-substituted-acetoxy)methyl]-2-furfuryl]dialkylamines. J. Med. Chem. 1994, 37, 4278–4287. 10.1021/jm00051a004. 7996539

[ref26] Plitta B.; Adamska E.; Giel-Pietraszuk M.; Fedoruk-Wyszomirska A.; Naskręt-Barciszewska M.; Markiewicz W. T.; Barciszewski J. New cytosine derivatives as inhibitors of DNA methylation. Eur. J. Med. Chem. 2012, 55, 243–254. 10.1016/j.ejmech.2012.07.024. 22854677

[ref27] Zhu M.-M.; Tao L.; Zhao Q.; Dong J.; Liu Y.-M.; He H.-Y.; Cao Y. Versatile CO-assisted direct reductive amination of 5-hydroxymethylfurfural catalyzed by a supported gold catalyst. Green Chem. 2017, 19, 3880–3887. 10.1039/C7GC01579H.

[ref28] García-Ortiz A.; Vidal J. D.; Climent M. J.; Concepción P.; Corma A.; Iborra S. Chemicals from biomass: Selective synthesis of N-substituted furfuryl amines by the one-pot direct reductive amination of furanic aldehydes. ACS Sustainable Chem. Eng. 2019, 7, 6243–6250. 10.1021/acssuschemeng.8b06631.

[ref29] Nuzhdin A. L.; Bukhtiyarova M. V.; Bukhtiyarov V. I. Two-step one-pot reductive amination of furanic aldehydes using CuAlO_x_ catalyst in a flow reactor. Molecules 2020, 25, 4771 10.3390/molecules25204771. 33080807 PMC7594031

[ref30] Ansorge-Schumacher M. B.; Thum O. Immobilised lipases in the cosmetics industry. Chem. Soc. Rev. 2013, 42, 6475–6490. 10.1039/C3CS35484A. 23515487

[ref31] Contesini F. J.; Davanço M. G.; Borin G. P.; Vanegas K. G.; Cirino J. P. G.; de Melo R. R.; Mortensen U. H.; Hildén K.; Campos D. R.; Carvalho P. O. Advances in recombinant lipases: Production, engineering, immobilization and application in the pharmaceutical industry. Catalysts 2020, 10, 1032 10.3390/catal10091032.

[ref32] Reyes-Reyes A. L.; Barranco F. V.; Sandoval G. Recent advances in lipases and their applications in the food and nutraceutical industry. Catalysts 2022, 12, 960 10.3390/catal12090960.

[ref33] Krystof M.; Pérez-Sánchez M.; Domínguez de María P. Lipase-catalyzed (trans)esterification of 5-hydroxymethylfurfural and separation from HMF esters using deep-eutectic solvents. ChemSusChem 2013, 6, 630–634. 10.1002/cssc.201200931. 23456887

[ref34] Krystof M.; Pérez-Sánchez M.; Domínguez de María P. Lipase-mediated selective oxidation of furfural and 5-hydroxymethylfurfural. ChemSusChem 2013, 6, 826–830. 10.1002/cssc.201200954. 23576295

[ref35] Qin Y.-Z.; Zong M.-H.; Lou W.-Y.; Li N. Biocatalytic upgrading of 5-hydroxymethylfurfural (HMF) with levulinic acid to HMF levulinate in biomass-derived solvents. ACS Sustainable Chem. Eng. 2016, 4, 4050–4054. 10.1021/acssuschemeng.6b00996.

[ref36] Stensrud K.; Smith B.; Archer Daniels Midland Company, Chicago, USA. Preparation of a sugar-derived ester, glycol and polymers therefrom. WO2017/065980A1, 2017.

[ref37] Uribe J.; Lienqueo M. E.; Guajardo N. Optimization and determination of kinetic parameters of the synthesis of 5-lauryl-hydroxymethylfurfural catalyzed by lipases. Catalysts 2023, 13, 19 10.3390/catal13010019.

[ref38] Stensrud K.; Wicklund L.; Archer Daniels Midland Company, Chicago, USA. Synthesis of non-ionic surfactants from 5-hydroxymethyl-2-furfural, furan-2,5-dimethanol and bis-2,5-dihydroxymethyl-tetrahydrofurans. US2017/0226075A1, 2017.

[ref39] Lăcătuş M. A.; Bencze L. C.; Toşa M. I.; Paizs C.; Irimie F. D. Eco-friendly enzymatic production of 2,5-bis(hydroxymethyl)furan fatty acid diesters, potential biodiesel additives. ACS Sustainable Chem. Eng. 2018, 6, 11353–11359. 10.1021/acssuschemeng.8b01206.

[ref40] Baraldi S.; Fantin G.; Di Carmine G.; Ragno D.; Brandolese A.; Massi A.; Bortolini O.; Marchetti N.; Giovannini P. P. Enzymatic synthesis of biobased aliphatic–aromatic oligoesters using 5,5′-bis(hydroxymethyl)furoin as a building block. RSC Adv. 2019, 9, 29044–29050. 10.1039/C9RA06621G. 35528403 PMC9071804

[ref41] Arias K. S.; Carceller J. M.; Climent M. J.; Corma A.; Iborra S. Chemoenzymatic synthesis of 5-hydroxymethylfurfural (HMF)-derived plasticizers by coupling HMF reduction with enzymatic esterification. ChemSusChem 2020, 13, 1864–1875. 10.1002/cssc.201903123. 31944622

[ref42] Lăcătuş M. A.; Dudu A. I.; Bencze L. C.; Katona G.; Irimie F.-D.; Paizs C.; Toşa M. I. Solvent-free biocatalytic synthesis of 2,5-bis-(hydroxymethyl)furan fatty acid diesters from renewable resources. ACS Sustainable Chem. Eng. 2020, 8, 1611–1617. 10.1021/acssuschemeng.9b06442.

[ref43] Gotor-Fernández V.; Gotor V. Enzymatic aminolysis and ammonolysis processes in the preparation of chiral nitrogenated compounds. Curr. Org. Chem. 2006, 10, 1125–1143. 10.2174/138527206777698084.

[ref44] Lima R. N.; dos Anjos C. S.; Orozco E. V. M.; Porto A. L. M. Versatility of *Candida antarctica* lipase in the amide bond formation applied in organic synthesis and biotechnological processes. Mol. Catal. 2019, 466, 75–105. 10.1016/j.mcat.2019.01.007.

[ref45] Ortiz C.; Ferreira M. L.; Barbosa O.; dos Santos J. C. S.; Rodrigues R. C.; Berenguer-Murcia Á.; Briand L. E.; Fernandez-Lafuente R. Novozym 435: The “perfect” lipase immobilized biocatalyst?. Catal. Sci. Technol. 2019, 9, 2380–2420. 10.1039/C9CY00415G.

[ref46] Cassimjee K. E.; Hendil-Forssell P.; Volkov A.; Krog A.; Malmo J.; Aune T. E. V.; Knecht W.; Miskelly I. R.; Moody T. S.; Humble M. S. Streamlined preparation of immobilized *Candida antarctica* lipase B. ACS Omega 2017, 2, 8674–8677. 10.1021/acsomega.7b01510. 30023589 PMC6045393

[ref47] Iglesias L. E.; Sánchez V. M.; Rebolledo F.; Gotor V. *Candida antarctica* B lipase catalysed resolution of (±)-l-(heteroaryl)ethylamines. Tetrahedron: Asymmetry 1997, 8, 2675–2677. 10.1016/S0957-4166(97)00330-3.

[ref48] Brem J.; Bencze L.-C.; Liljeblad A.; Turcu M. C.; Paizs C.; Irimie F.-D.; Kanerva L. T. Chemoenzymatic preparation of 1-heteroarylethanamines of low solubility. Eur. J. Org. Chem. 2012, 2012, 3288–3294. 10.1002/ejoc.201200330.

[ref49] Blume F.; Albeiruty M. H.; Deska J. Alkylative amination of biogenic furans through imine-to-azaallyl anion umpolung. Synthesis 2015, 47, 2093–2099. 10.1055/s-0034-1380201.

[ref50] Alcántara A. R.; Domínguez de María P. Recent advances on the use of 2-methyltetrahydrofuran (2-MeTHF) in biotransformations. Curr. Green Chem. 2018, 5, 86–103. 10.2174/2213346105666180727100924.

[ref51] Piazzolla F.; Temperini A. Recent advances in chemoselective acylation of amines. Tetrahedron Lett. 2018, 59, 2615–2621. 10.1016/j.tetlet.2018.05.065.

[ref52] Le Joubioux F.; Henda Y. B.; Bridiau N.; Achour O.; Graber M.; Maugard T. The effect of substrate structure on the chemoselectivity of *Candida antarctica* lipase B-catalyzed acylation of amino-alcohols. J. Mol. Catal. B: Enzym. 2013, 85–86, 193–199. 10.1016/j.molcatb.2012.09.006.

[ref53] Le Joubioux F.; Bridiau N.; Henda Y. B.; Achour O.; Graber M.; Maugard T. The control of Novozym 435 chemoselectivity and specificity by the solvents in acylation reactions of amino-alcohols. J. Mol. Catal. B: Enzym. 2013, 95, 99–110. 10.1016/j.molcatb.2013.06.002.

[ref54] Sheldon R. A. The *E* factor 25 years on: The rise of green chemistry and sustainability. Green Chem. 2017, 19, 18–43. 10.1039/C6GC02157C.

