# Supplementary material for: Chemoselective Lipase-Catalyzed Synthesis of Amido Derivatives from 5-Hydroxymethylfurfurylamine
Source: ACS Sustain Chem Eng. 2023 Jul 6;11(28):10284–92. doi: 10.1021/acssuschemeng.3c00775 (PMC10354804; doi:10.1021/acssuschemeng.3c00775)
Supplement: Supplementary file 1 — sc3c00775_si_001.pdf [file sc3c00775_si_001.pdf]

Chemoselective Lipase-Catalyzed Synthesis of Amido Derivatives from 5-Hydroxymethylfurfurylamine

Antía Pintor,[1,2] *Iván Lavandera,*[1] *Alexey Volkov,**[2] *and Vicente Gotor-Fernández**[1]

[1] Organic and Inorganic Chemistry Department, University of Oviedo. Avenida Julián Clavería 8. Oviedo 33006 (Spain).

[2] EnginZyme AB. Tomtebodavägen 6. 171 65 Solna (Sweden).

A.V.: Phone number: +46736846899. E-mail: alexey@enginzyme.com

V.G.-F.: Phone number: (+34)985103454. E-mail: vicgotfer@uniovi.es

Number of pages: 33

Number of figures: 5 (Scheme S1 and Figures S1 to S4)

Number of tables: 4 (Tables S1 to S4)

Index

1. Chemical synthesis of *N*-(furan-2-ylmethyl)acetamide (6) **S3**

2. Lipase-catalyzed acetylation of amine 4 .... **S3**

2.1. General protocol for the lipase screening in the acetylation of amine 4 ..... **S3**

2.2. Monitorization of the CALB-catalyzed acetylation of amine 4 .... **S4**

2.3. Solvent screening for the EziG-CALB-catalyzed acetylation of amine 4 .... **S5**

3. Acetylation of HMFA (3) using different lipases and acyl donors **S5**

3.1. Lipase screening for the acetylation of HMFA with EtOAc **S5**

3.2. Acylation of HMFA with EziG-CALB using different acyl donors 5a-p **S5**

3.3. Study of the influence of the HMFA concentration using EtOAc **S7**

4. Environmental assessment of the enzymatic acetylation **S8**

5. Analytical methods (Gas chromatography analyses) **S9**

6. NMR spectra **S10**

1. Chemical synthesis of *N*-(furan-2-ylmethyl)acetamide (6)

Before studying the lipase-catalyzed acylation of HMF (**3**), 2-furfurylamine (**4**) was selected as a model substrate due to its commercial availability at low prize. The chemical synthesis of the corresponding acetamide, namely *N*-(furan-2-ylmethyl)acetamide (**6**), was initially performed to develop adequate GC methods to follow later the enzymatic reactions (see Section 4). The experimental protocol was as follows (Scheme S1): Acetic anhydride (Ac_2O, 389 µL, 4.12 mmol, 4 equiv) and triethylamine (Et_3N, 377 µL, 2.68 mmol, 2.6 equiv) were successively added to a solution of commercial 2-furfurylamine (**4**, 100 mg, 1.03 mmol, 200 mM) in dry dichloromethane (5.15 mL). The reaction was stirred at room temperature (rt) after complete consumption of the starting amine (22 h). After this time the reaction mixture was quenched with water (10 mL), extracted with CH_2Cl_2 (3 x 10 mL), dried over Na_2SO_4, filtered and the solvent evaporated under reduced pressure. The reaction crude was purified by column chromatography on silica gel (eluent gradient from 40 to 50% EtOAc/Hexane), yielding *N*-(furan-2-ylmethyl)acetamide (**6**) in 78% isolated yield (139.1 mg).

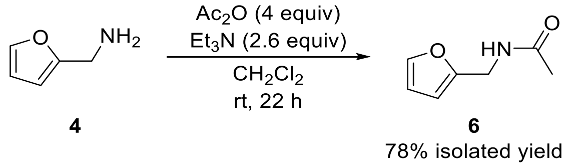

Scheme S1. Chemical synthesis of *N*-(furan-2-ylmethyl)acetamide.

2. Lipase-catalyzed acetylation of amine 4

The lipase-catalyzed acetylation of 2-furfurylamine (**4**) was developed with different hydrolases (see main text for additional information), prior developing the optimization of the reaction conditions.

2.1. General protocol for the lipase screening in the acetylation of amine 4

Amine **4** (20 mg, 0.2 mmol, 100 mM) was dissolved in methyl *tert*-butyl ether (MTBE, 2 mL) in an Erlenmeyer-flask, then the corresponding hydrolase (20 mg, 1:1 w/w enzyme:**4** ratio) and EtOAc (59 µL, 0.6 mmol, 3 equiv) were successively added. The reaction was shaken at 250 rpm for 24 h at 30 °C, and after this time an aliquot was taken and analyzed by GC (see Section 4). The conversion values are depicted in Table S1.

Table S1. Screening of lipases for the acetylation of **4** with EtOAc as acyl donor.

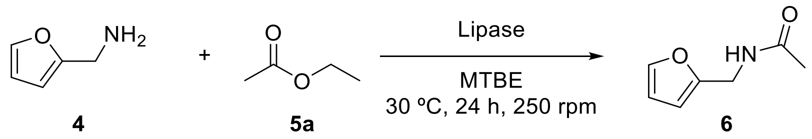

Entry	Lipase	Conversion (%)[a]
1	*Candida antarctica* lipase type B (Novozyme 435®)	>99
2	*Candida antarctica* lipase type B (EziG-CALB)	>99
3	*Thermomyces lanuginosus* lipase (TLL)	83
4	*Pseudomonas cepacia* lipase (PSL)	69
5	Lipase AK from *Pseudomonas fluorescens* (AKL)	31
6	*Candida rugosa* lipase (CRL)	6
7	*Candida antarctica* lipase type A (CALA)	4
8	*Aspergillus niger* lipase (ANL)	2

[a] Conversion values were determined by GC analyses of the reaction crude.

2.2. Monitorization of the CALB-catalyzed acetylation of amine 4

Both immobilized CALB forms, the one from Novozymes and the one from EnginZyme were used for the acetylation of amine **4**, and a reaction time course study was performed, taking regular aliquots after 0.5, 1 and 2 h (Figure S1).

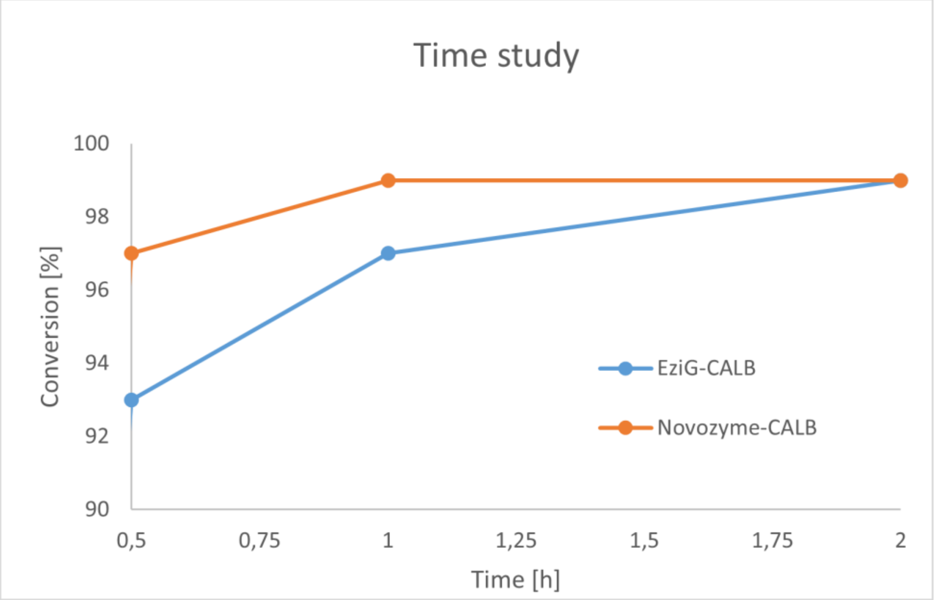

Figure S1. Time course study for the acetylation of 2-furfurylamine (**4**, 20 mg, 100 mM) using immobilized CALB (Novozyme 435® or EziG-CALB, 20 mg), EtOAc (3 equivalents) in MTBE at 30 °C and 250 rpm. Conversion values were determined by GC analyses of the reaction crudes.

2.3. Solvent screening for the EziG-CALB-catalyzed acetylation of amine 4

The lipase-catalyzed acetylation of amine **4** was developed following the general protocol described in Section 2.1, but during only 2 h and using different solvents as disclosed in Table S2.

Table S2. Screening of solvents for the acetylation of **4** using EziG-CALB.[a]

Entry	Solvent	6 (%)[b]
1	MTBE	>99
2	Et_2O	>99
3[c]	EtOAc	>99
4	2-MeTHF	99
5	1,4-Dioxane	96
6	Toluene	84
7	Tetrahydrofuran	72
8	Hexane	10

[a] Reaction conditions: **4** (20 mg, 100 mM), 3 equiv of **5a** and EziG-CALB (20 mg, 1:1 w/w enzyme:**4** ratio) for 2 h at 30 ºC and 250 rpm.
[b] Product percentage calculated by GC analyses.
[c] Used as solvent and acyl donor.

3. Acetylation of HMFA (3) using different lipases and acyl donors

3.1. Lipase screening for the acetylation of HMFA with EtOAc

HMFA (**3**, 15 mg, 0.12 mmol, 100 mM) was dissolved in 2-MeTHF (1.2 mL) inside an Erlenmeyer-flask, then the corresponding hydrolase (15 mg, 1:1 w/w enzyme:**3** ratio) and EtOAc (**5a**, 15 µL, 0.15 mmol, 1.3 equiv) were successively added. The reaction was shaken at 250 rpm for 24 h at 30 °C, and after that time an aliquot was taken and analyzed by GC (see Section 4). The conversion values are depicted in Table 1 of the manuscript.

3.2. Acylation of HMFA with EziG-CALB using different acyl donors 5a-p

Lipase-catalyzed selective N-acylation was performed using EziG-CALB under optimized reaction conditions (Table S3, see experimental section of the manuscript for detailed information).

Table S3. Acyl donor study for the acylation of HMFA with EziG-CALB in 2-MeTHF.

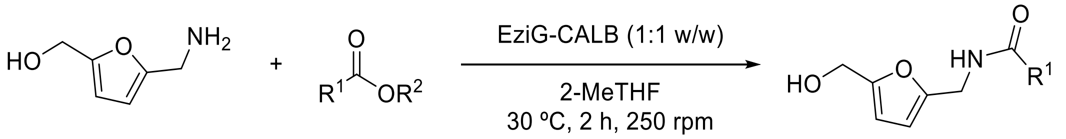

3 (100 mM) | **5a-p** (1.3 equiv) | **7a-i**

Entry	Acyl donor 5a-p	R[1]	R[2]	7a-i (%)[a]
1	**a**	CH_3	CH_3CH_2	>99 (**7a**)
2	**b**	CH_3OCH_2	CH_3CH_2	>99 (**7b**)
3	**c**	CH_3CH_2	CH_3CH_2	>99 (**7c**)
4	**d**	$ClCH_2$	CH_3CH_2	>99 (**7d**)
5	**e**	$PhCH_2$	CH_3CH_2	>99 (**7e**)
6	**f**	CH_3	CH_3	>99 (**7a**)
7	**g**	CH_3	$CH_3CH_2CH_2$	>99 (**7a**)
8	**h**	CH_3	$CH_3CH_2CH_2CH_2$	>99 (**7a**)
9	**i**	CH_3	$(CH_3)_2CH$	>99 (**7a**)
10	**j**	CH_3	$PhCH_2$	>99 (**7a**)
11	**k**	CH_3	4-NO_2-C_6H_4	>99 (**7a**)
12	**l**	CH_3OCH_2	CH_3	>99 (**7b**)
13	**m**	*n*-Propyl	CH_3	>99 (**7f**)
14	**n**	Pentyl	CH_3CH_2	>99 (**7g**)
15	**o**	Nonyl	CH_3CH_2	>99 (**7h**)
16	**p**	Undecyl	CH_3	>99 (**7i**)

[a] Percentage of products measured by ^{1}H-NMR analyses of the reaction crude. Quantitative isolated yields were reached after filtration and evaporation of the filtrate for most of the reactions. For ethyl phenylacetate (**5e**) the reaction crude was dried on the freeze-dryer overnight. For benzyl acetate (**5j**) and 4-nitrophenyl acetate (**5k**), the formation of product **7a** was observed in complete conversion, but no purification was performed to remove the remaining acyl donor. For ethyl caprate (**5o**) methyl laurate (**5p**), to remove the excess of the acyl donor, the reaction crude was washed with cold Et_2O (3 x 2 mL).

3.3. Study of the influence of the HMFA concentration using EtOAc

Ethyl acetate (1.3 equiv) was added to a mixture of HMFA (**3**, 30-150 mg, 0.24-1.2 mmol, 200-1000 mM), EziG-CALB (15 mg), and 2-MeTHF (1.2 mL). The corresponding mixture was shaken between 2 and 22 h at 250 rpm and 30 °C, taking aliquots regularly that were analyzed by GC (see Figure S2 and Table S4).

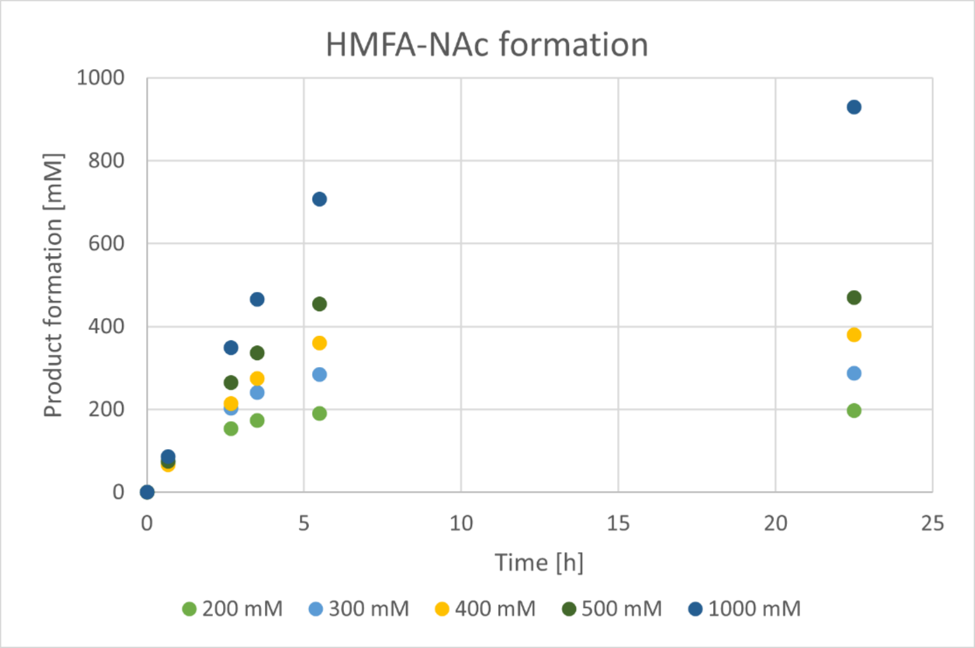

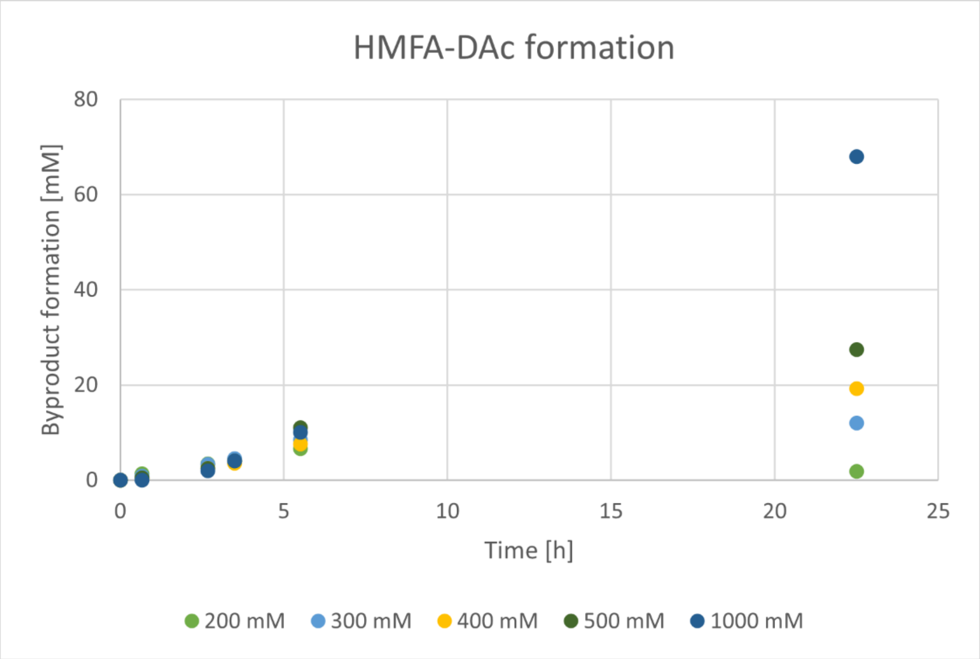

Figure S2. Time course study for the acetylation of **4** at different substrate concentrations (200-1000 mM) using immobilized EziG-CALB (15 mg), EtOAc (1.3 equivalents) in 2-MeTHF at 30 °C and 250 rpm (top) and concentration of diacetylated **8a** formed (bottom). Conversion values were determined by GC analyses.

4. Environmental assessment of the enzymatic acetylation

Environmental *E*-factor calculations (Figures S3 and S4) were performed based on the scale-up of the transformation at 1 g of substrate **3** (200 mM) with EtOAc as acylating agent using EziG-CALB. The transformation was treated as proceeding to the corresponding isolated yield, hence all losses in yield are accounted for as ‘material loss’.

	to obtain 1,13 g product (g)	to obtain 1 kg Amine (g)	to obtain 1 kg Amine (kg)	Waste (kg per Kg of P)	Waste (kg/Kg of P)-excl solv
Substrate (g)	1,00	885,0	0,88	0,00	0,00
EtOAc	0,69	613,3	0,61	0,00	0,00
EziG-CALB	0,50	442,5	0,44	0,44	0,44
2-MeTHF	32,71	28946,9	28,95	28,95	0,00
EtOAc (excess)	0,21	185,0	0,18	0,18	0,18
EtOH (co-product)	0,31	272,6	0,27	0,27	0,27
CH_2Cl_2 (wash)	26,60	23539,8	23,54	23,54	0,00
Material loss	0,20	177,0	0,18	0,18	0,18
			E-factor	53,6	1,1
	Upstream	Downstream	Upstream (Kg Kg^{-1})	Downstream (Kg Kg^{-1})	
EziG-CALB	0,44		0,8%		
2-MeTHF	28,95		54,0%		
EtOAc (excess)	0,18		0,3%		
EtOH (co-product)	0,27		0,5%		
CH_2Cl_2 (wash)		23,54		43,9%	
Material loss		0,18		0,3%	
E- factor contribution	55,7%	44,3%			
E- factor total	53,6				

Figure S3. Calculated *E*-factor for the acetylation of **3** with EtOAc using EziG-CALB at 1-g scale.

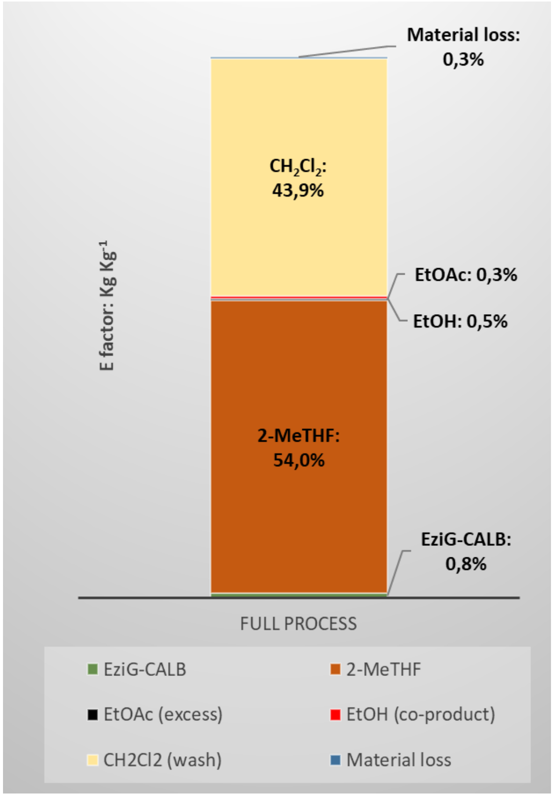

Figure S4. Contribution of the different components to the *E*-factor for the acetylation with EtOAc of **3** using EziG-CALB at 1-g scale.

5. Analytical methods (Gas chromatography analyses)

GC analyses were performed on an Agilent HP6890 GC chromatograph equipped with a FID detector. In all cases, a HP-1 column (30 m x 0.32 mm x 0.25 μm) was used for the determination of conversion values and product percentages (Table S4).

Table S4. GC analytical conditions and retention times for the determination of product percentages values.

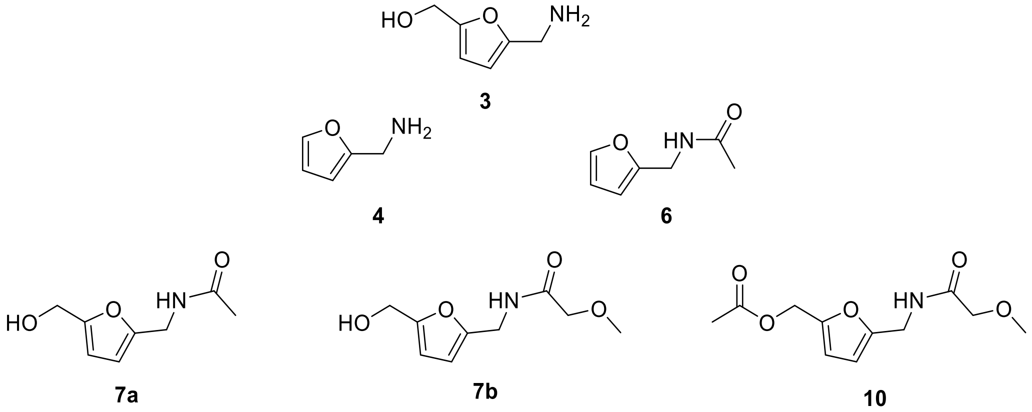

Entry	Compound	GC program[a]	Retention time (min)
1	**3**	60/2/15/225/5	6.2
2	**4**	60/2/15/225/5	2.4
3	**6**	60/2/15/225/5	6.7
4	**7a**	60/2/15/225/5	9.9
5	**7b**	60/2/15/225/5	10.7
6	**8a**	60/2/15/225/5	12.7
7	**10**	60/2/15/225/5	11.3

[a] GC program: initial temp. (ºC) / time (min) / ramp (ºC/min) / final temp. (ºC) / time (min).

6. NMR spectra

N-Acetyl-2-(aminomethyl)furan (6)

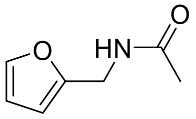

6

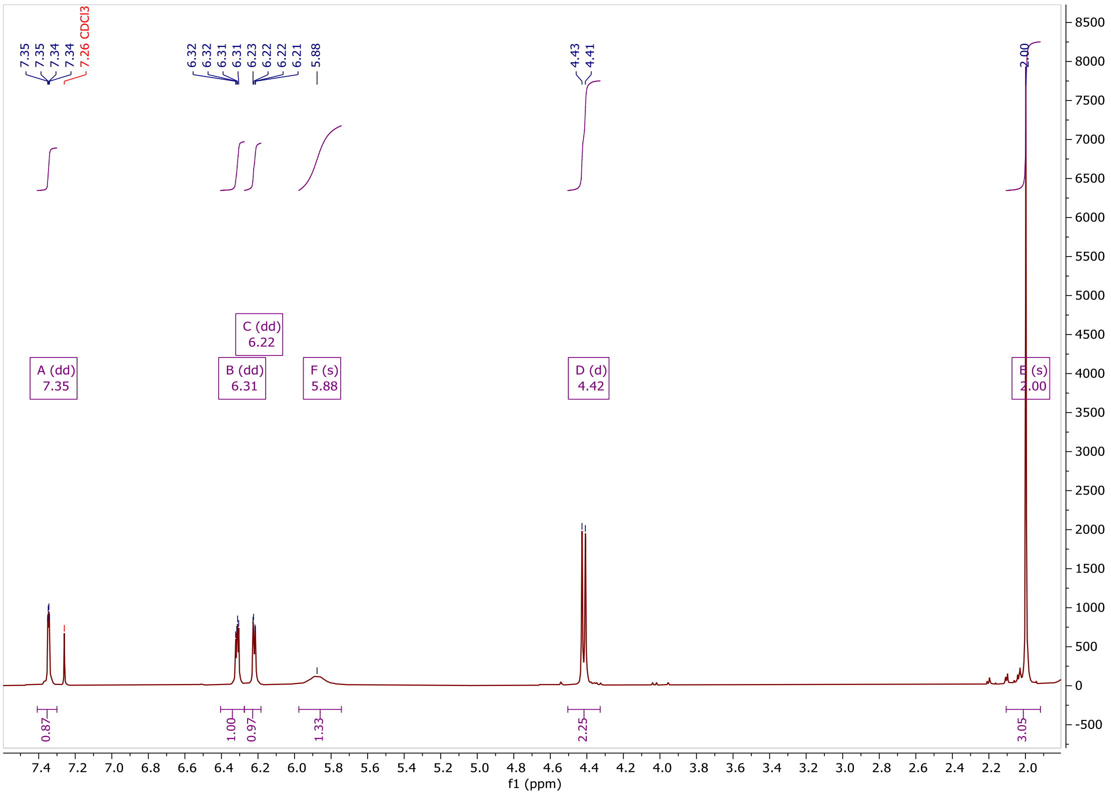

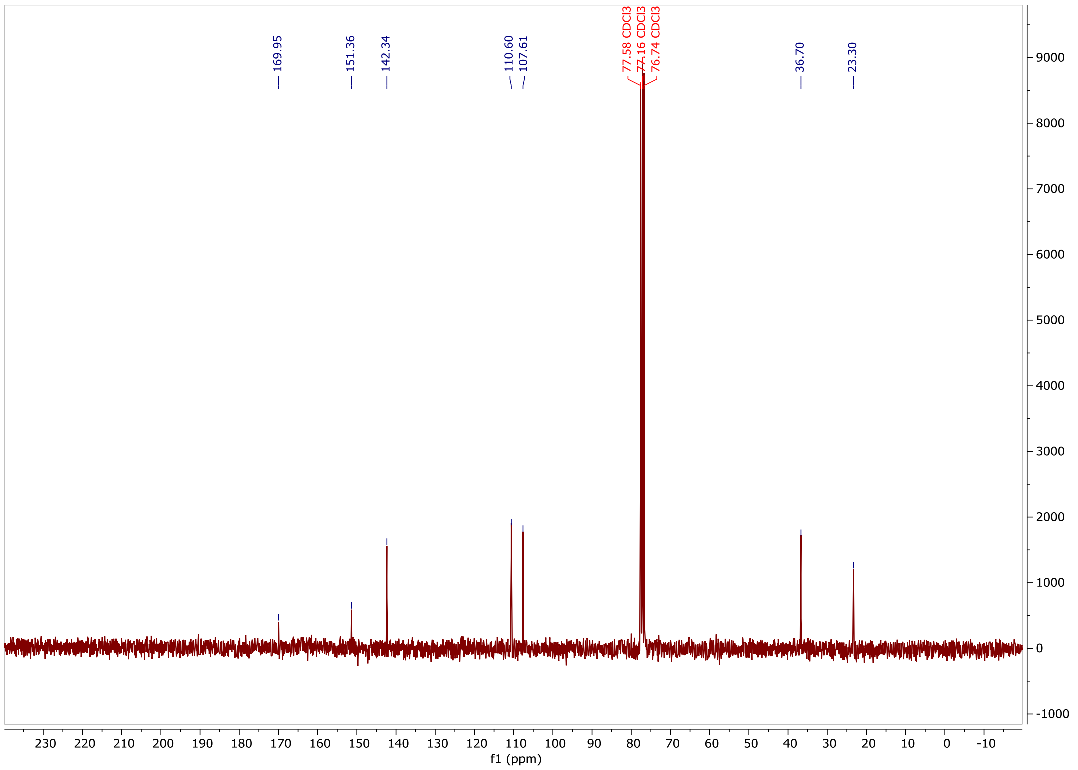
169.95
151.36
142.34
110.60
107.61
77.58 CDCl3
77.16 CDCl3
76.74 CDCl3
36.70
23.30
f1 (ppm)

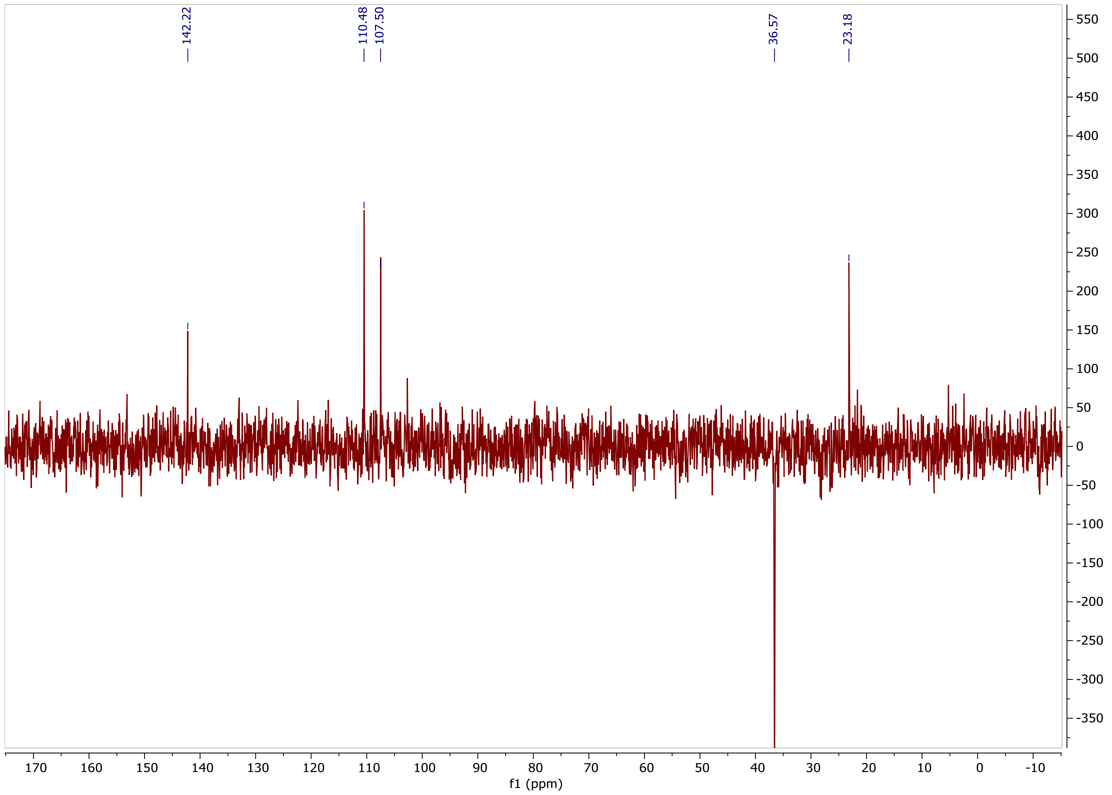
142.22
110.48
107.50
36.57
23.18
f1 (ppm)

N-[(5-(Hydroxymethyl)furan-2-yl)methyl]acetamide (7a)

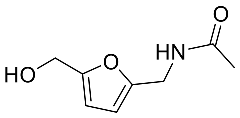

7a

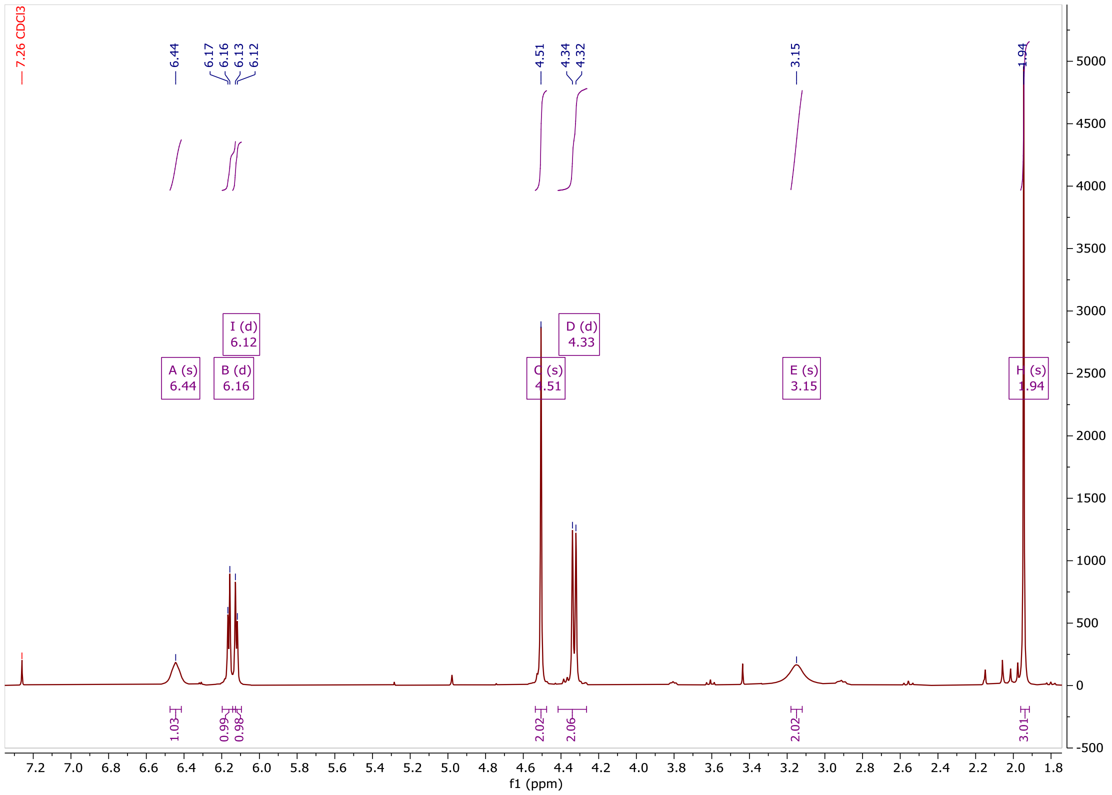

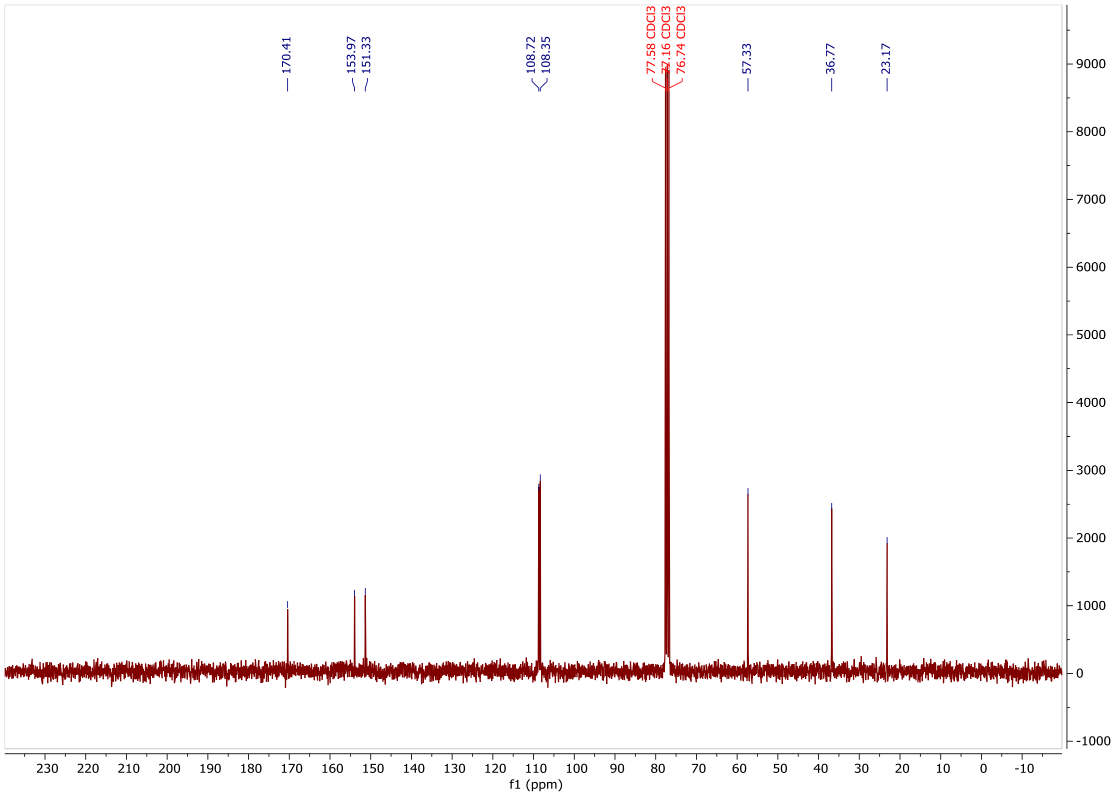

170.41
153.97
151.33
108.72
108.35
77.58 CDCl3
77.16 CDCl3
76.74 CDCl3
57.33
36.77
23.17
f1 (ppm)

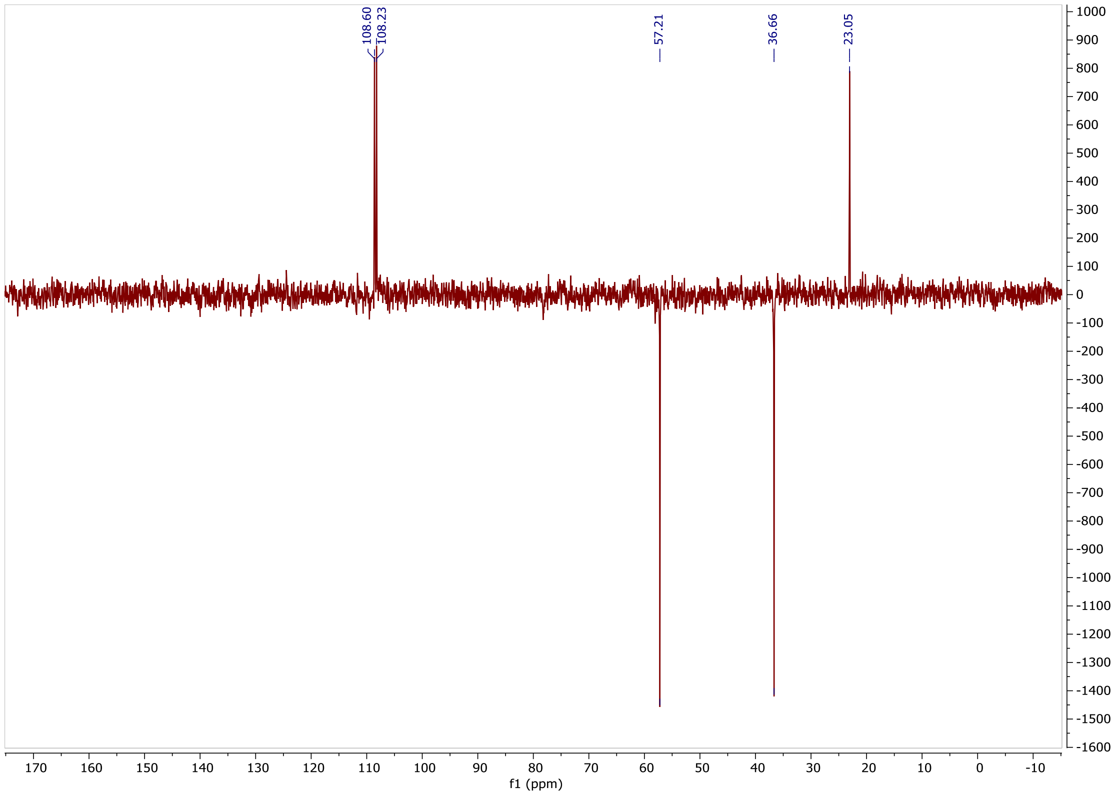

108.60
108.23
57.21
36.66
23.05
f1 (ppm)

[5-(Acetamidomethyl)furan-2-yl]methyl acetate (8a)

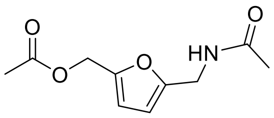

8a

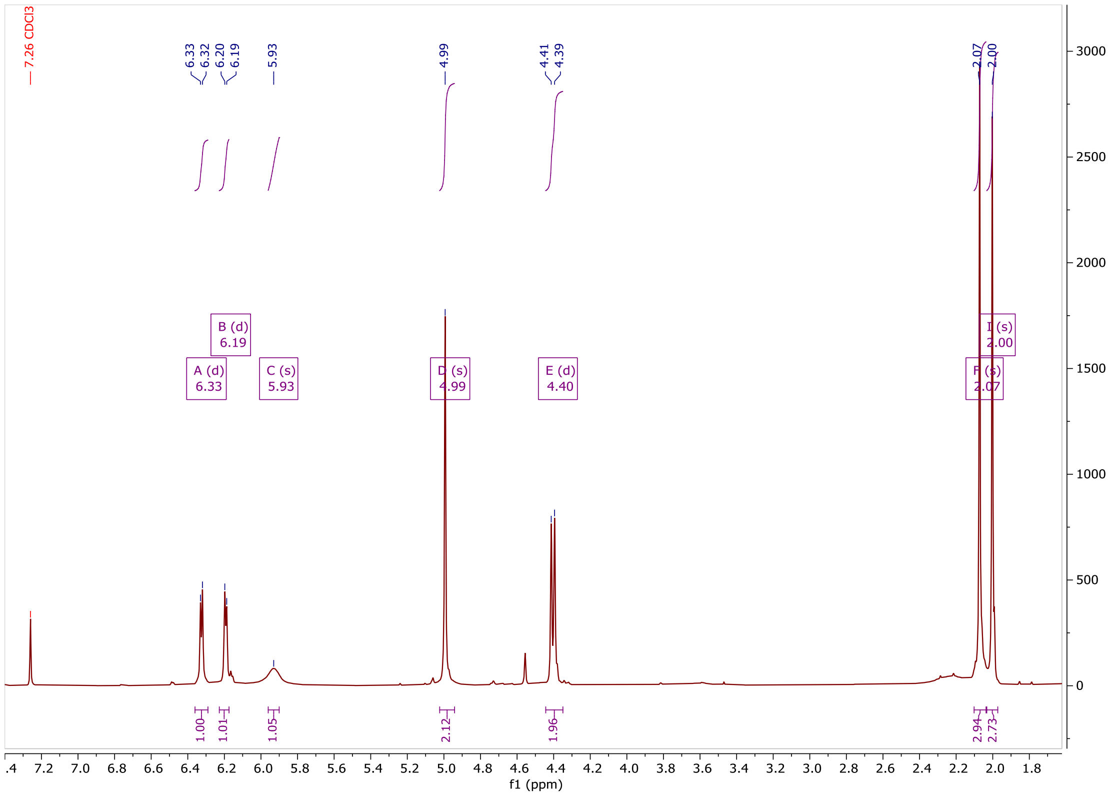

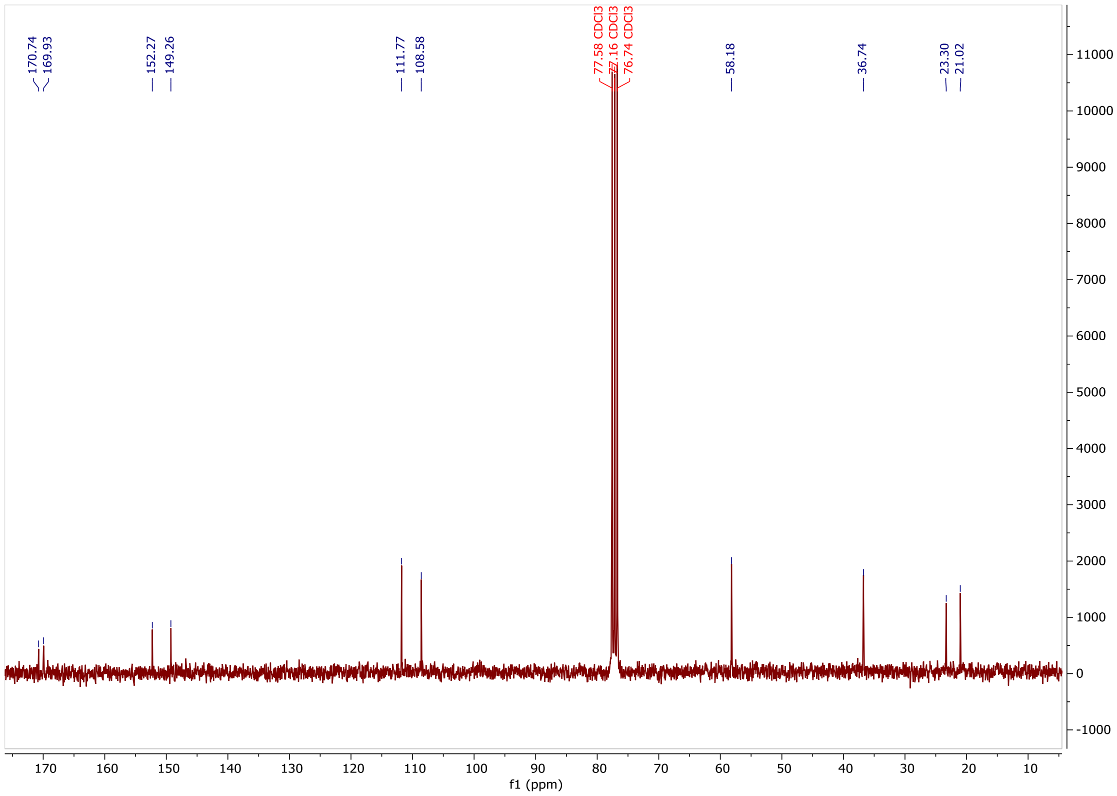

170.74
169.93
152.27
149.26
111.77
108.58
77.58 CDCl3
77.16 CDCl3
76.74 CDCl3
58.18
36.74
23.30
21.02
f1 (ppm)

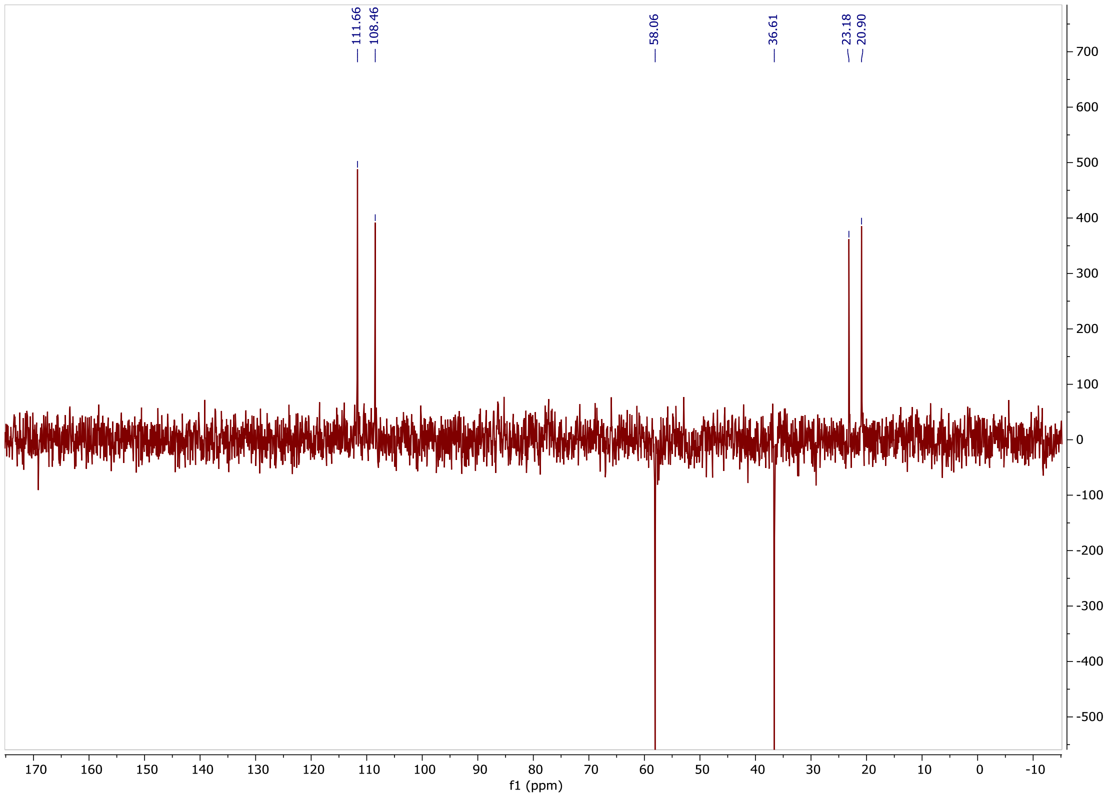

111.66
108.46
58.06
36.61
23.18
20.90
f1 (ppm)

N-[(5-(Hydroxymethyl)furan-2-yl)methyl]-2-methoxyacetamide (7b)

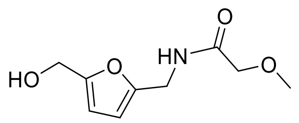

7b

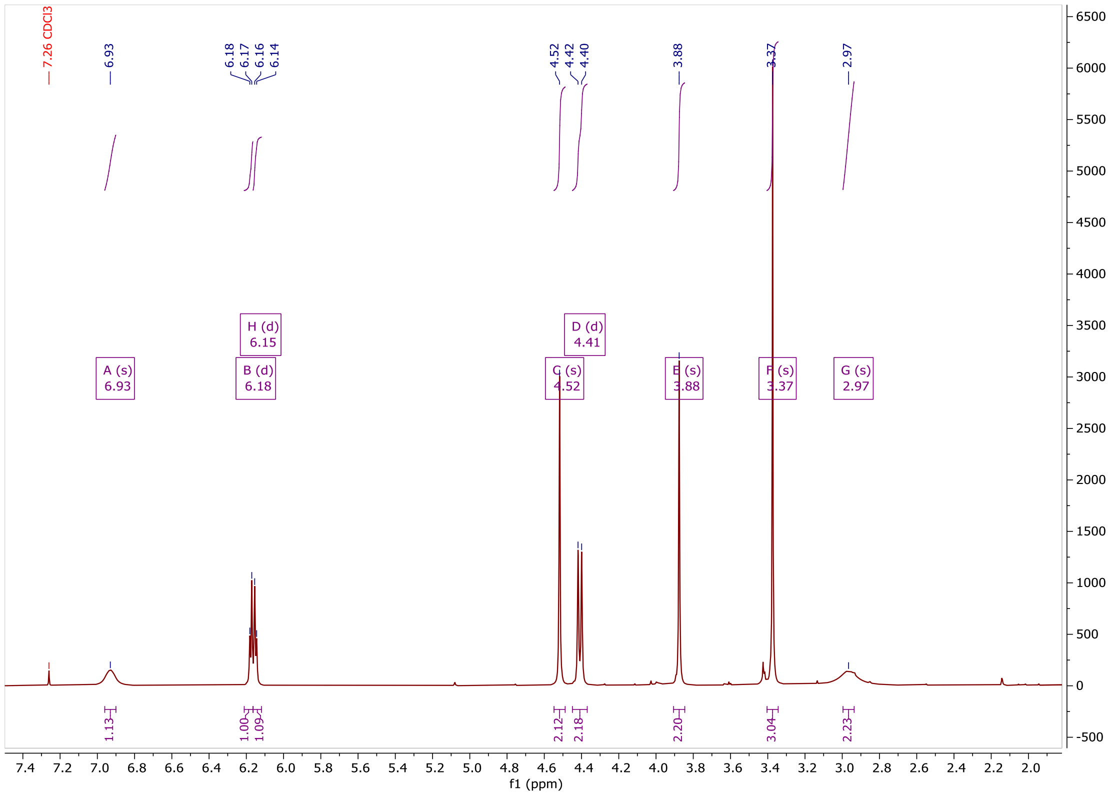

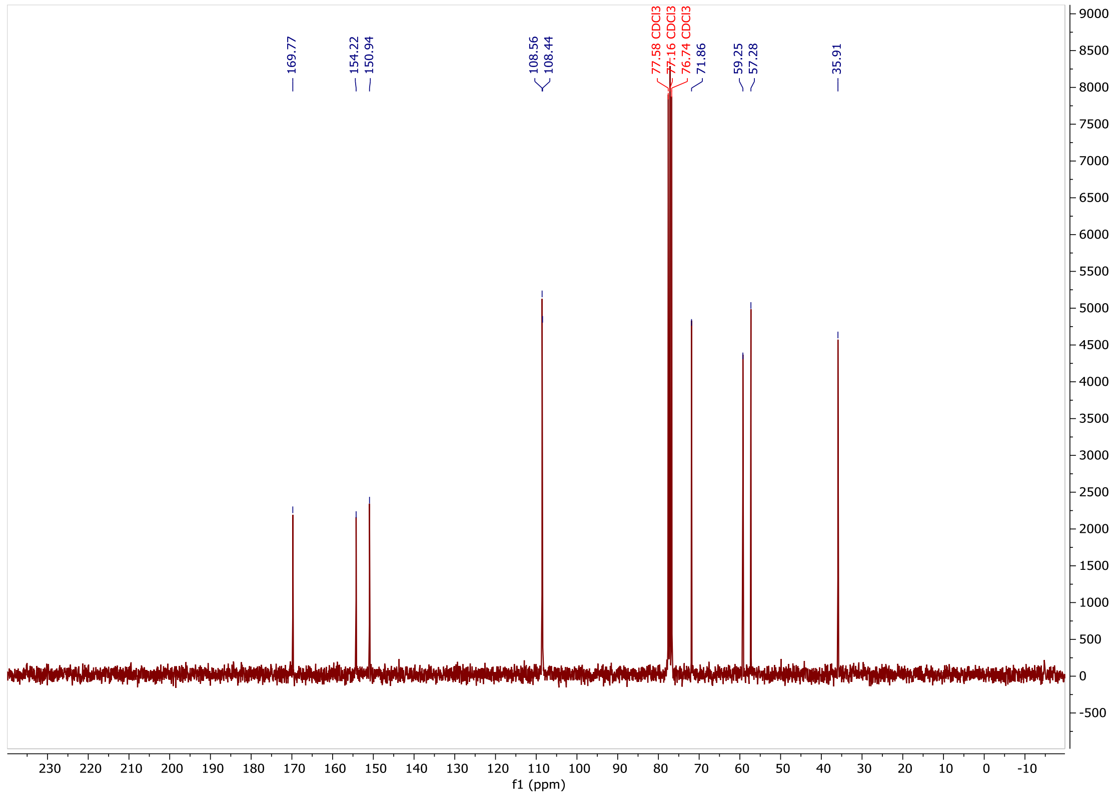
169.77
154.22
150.94
108.56
108.44
77.58 CDCl3
77.16 CDCl3
76.74 CDCl3
71.86
59.25
57.28
35.91
f1 (ppm)

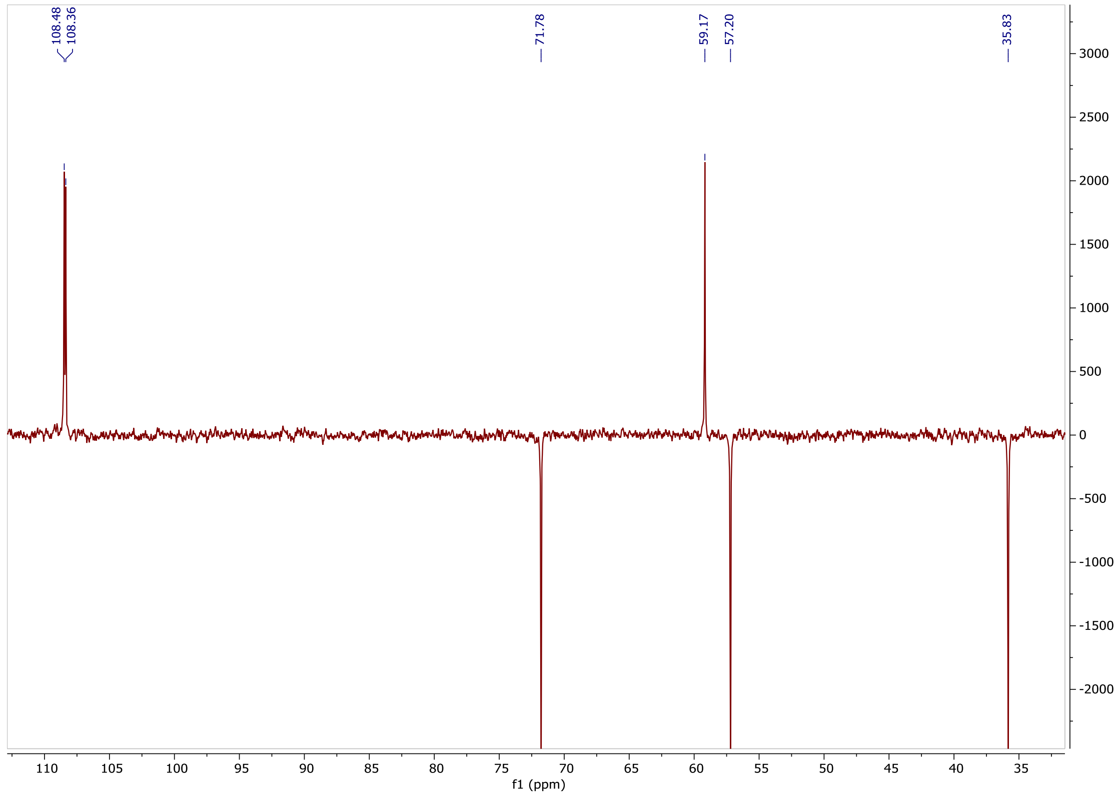
108.48
108.36
71.78
59.17
57.20
35.83
f1 (ppm)

N-[(5-(Hydroxymethyl)furan-2-yl)methyl]propionamide (7c)

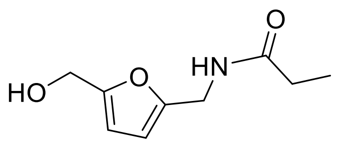

7c

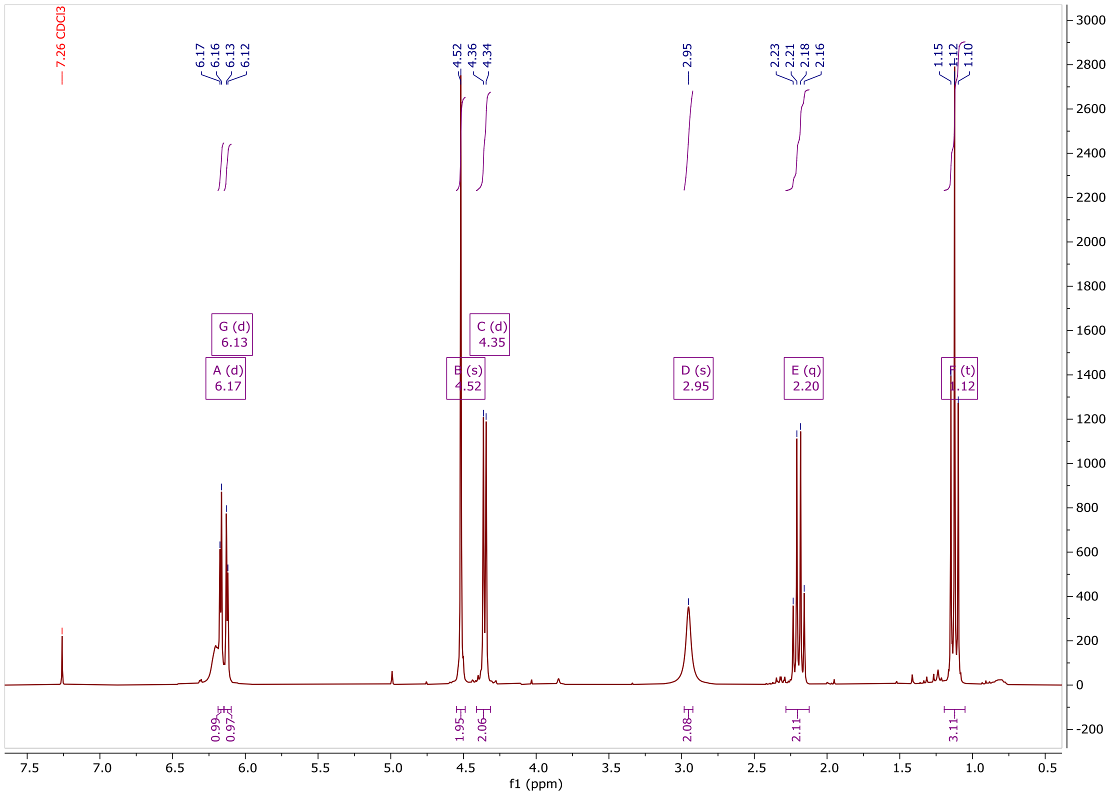

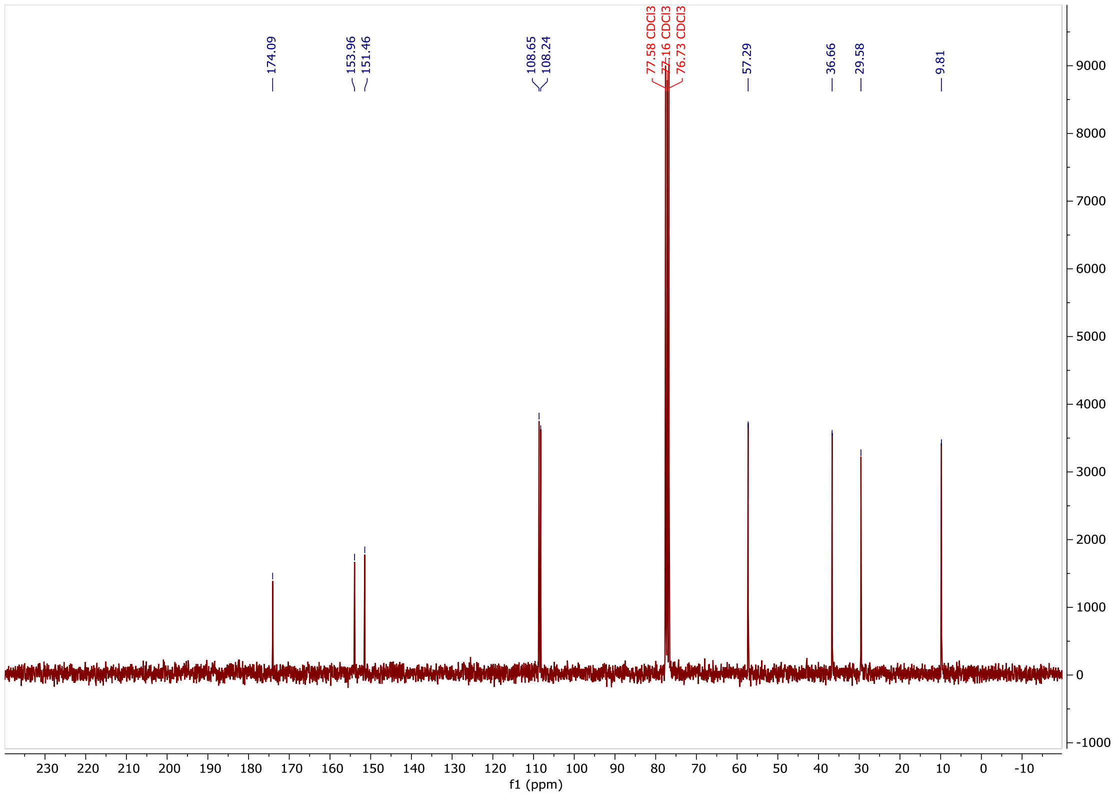
174.09
153.96
151.46
108.65
108.24
77.58 CDCl3
77.16 CDCl3
76.73 CDCl3
57.29
36.66
29.58
9.81
f1 (ppm)

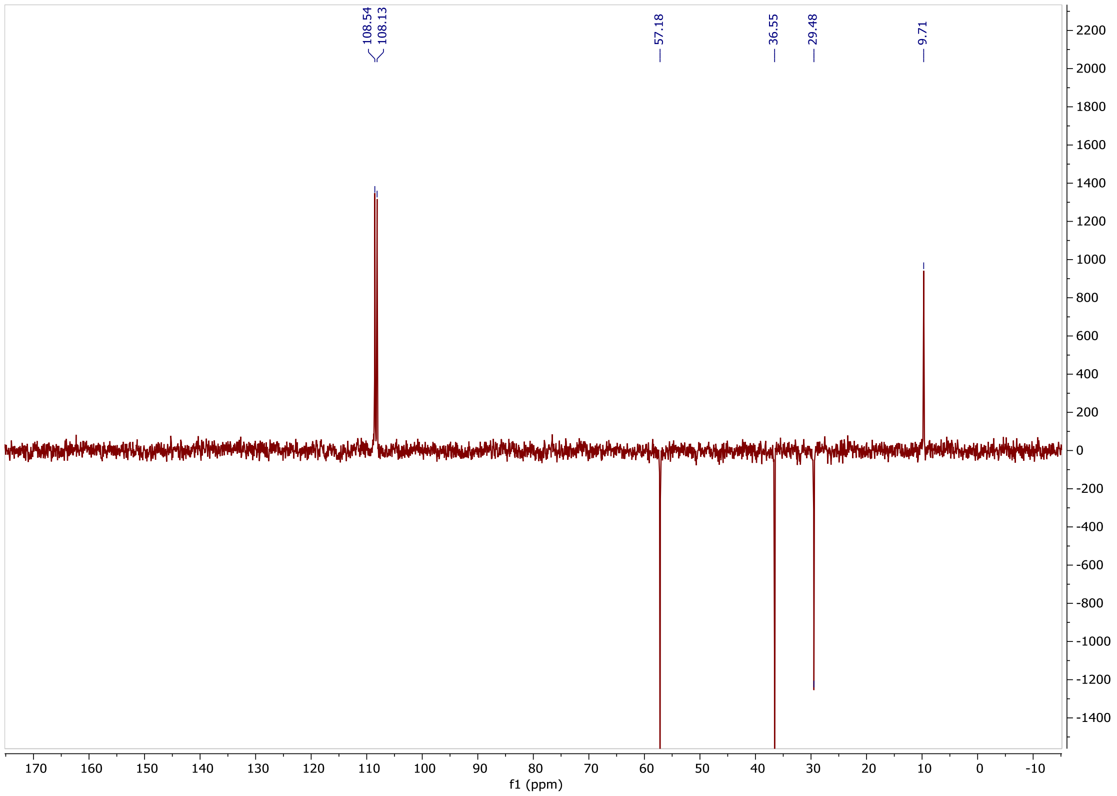
108.54
108.13
57.18
36.55
29.48
9.71
f1 (ppm)

2-Chloro-*N*-[(5-(hydroxymethyl)furan-2-yl)methyl]acetamide (7d)

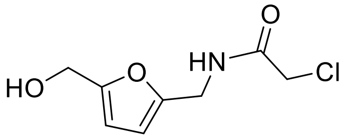

7d

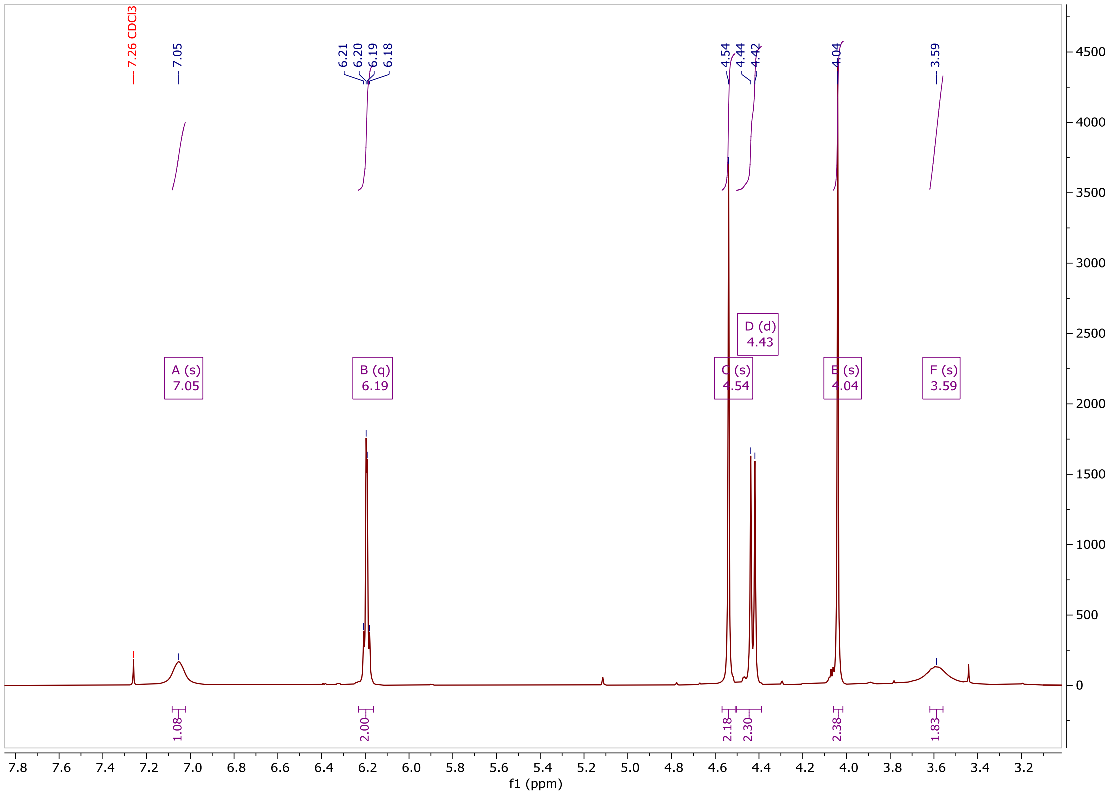

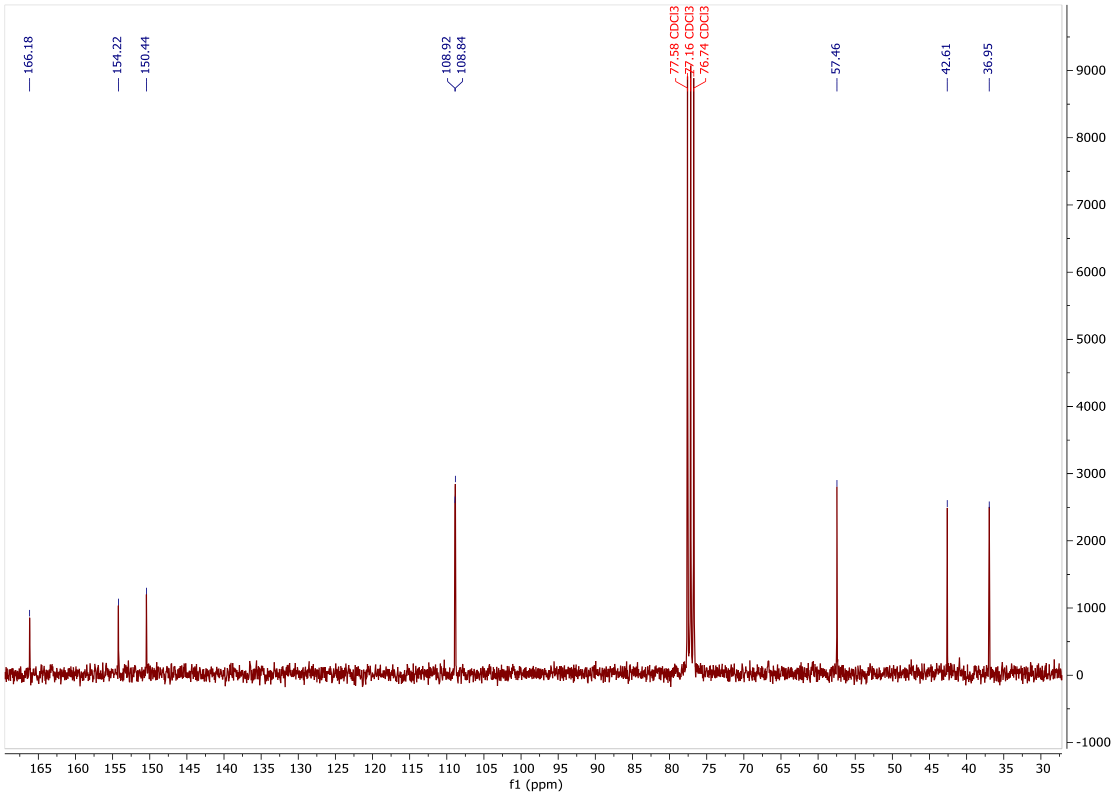

166.18
154.22
150.44
108.92
108.84
77.58 CDCl3
77.16 CDCl3
76.74 CDCl3
57.46
42.61
36.95
f1 (ppm)

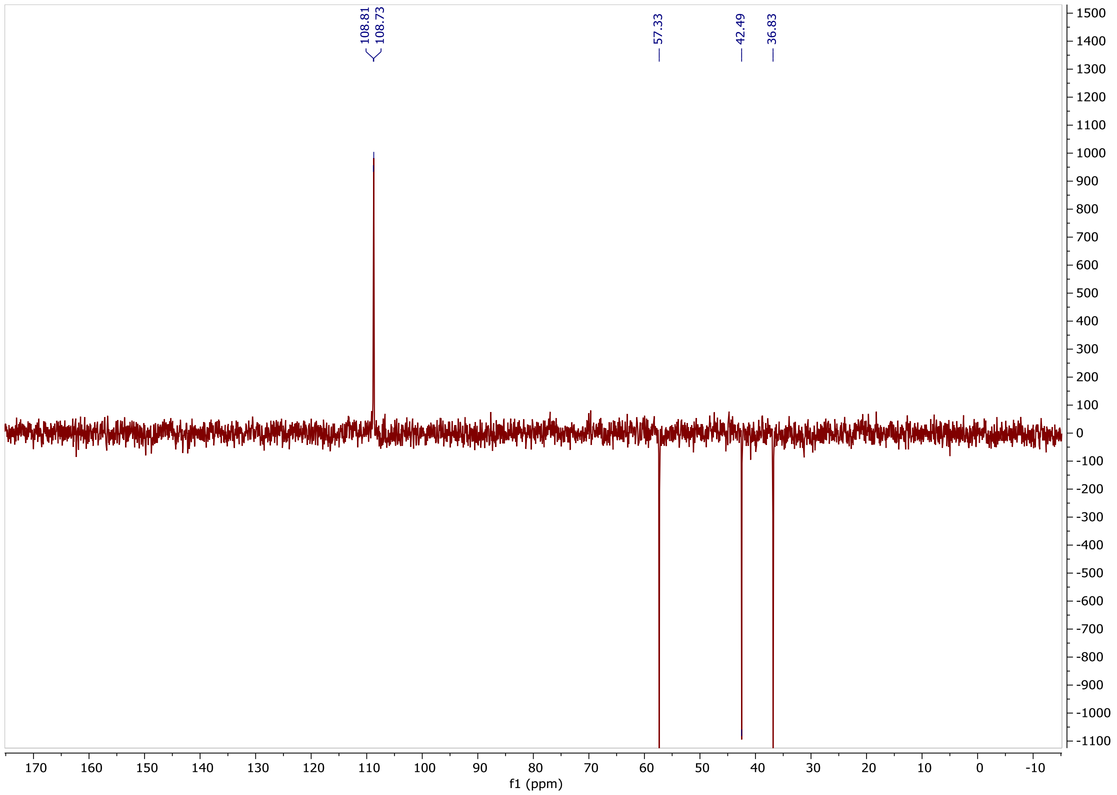

108.81
108.73
57.33
42.49
36.83
f1 (ppm)

N-[(5-(Hydroxymethyl)furan-2-yl)methyl]-2-phenylacetamide (7e)

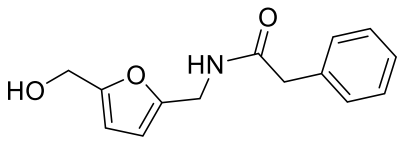

7e

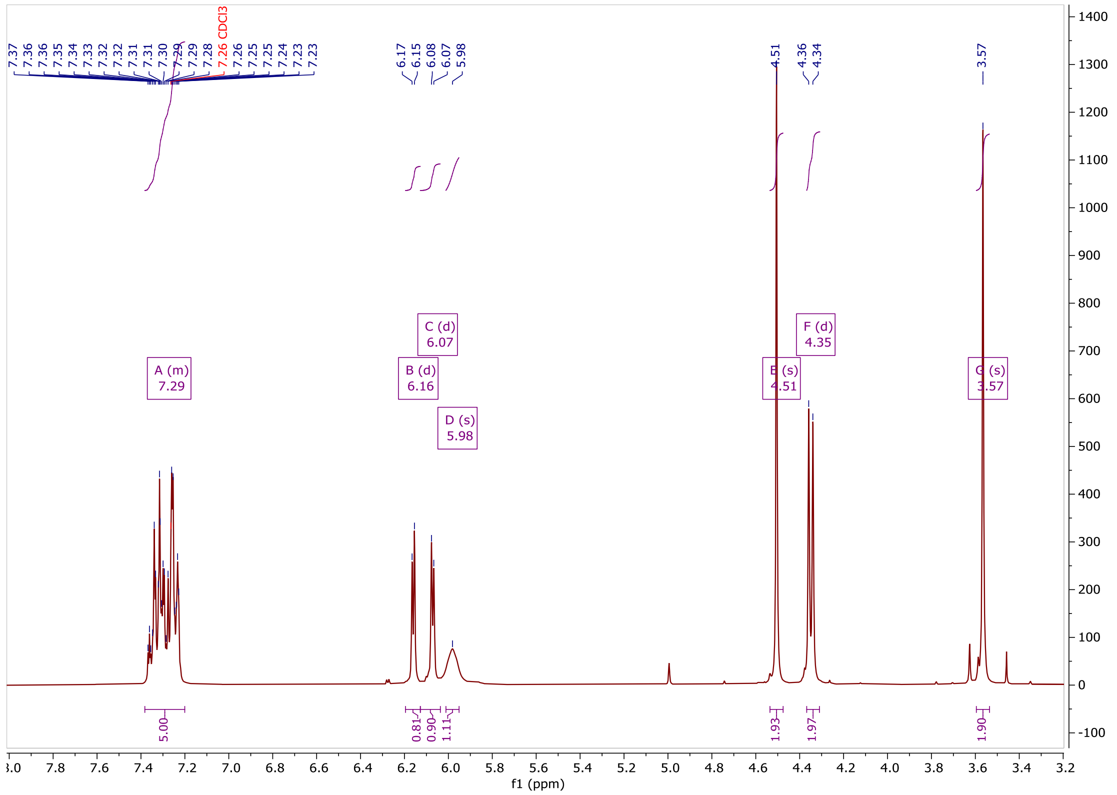

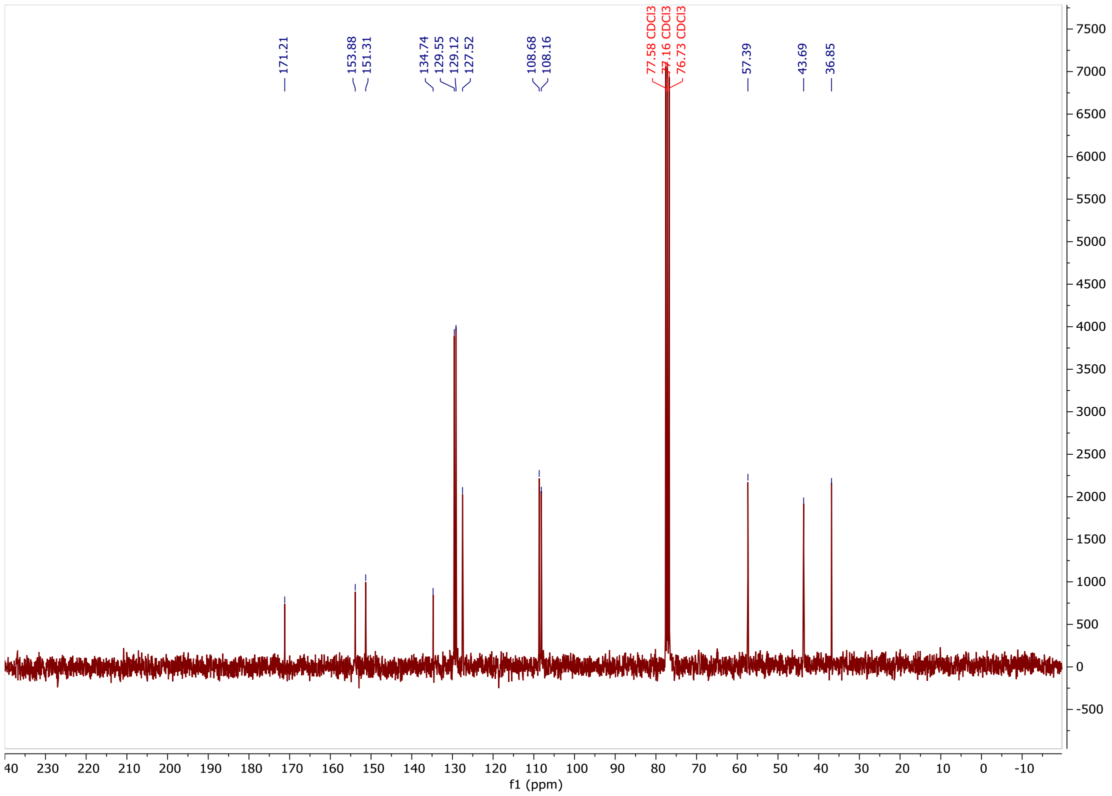

171.21
153.88
151.31
134.74
129.55
129.12
127.52
108.68
108.16
77.58 CDCl3
77.16 CDCl3
76.73 CDCl3
57.39
43.69
36.85
f1 (ppm)

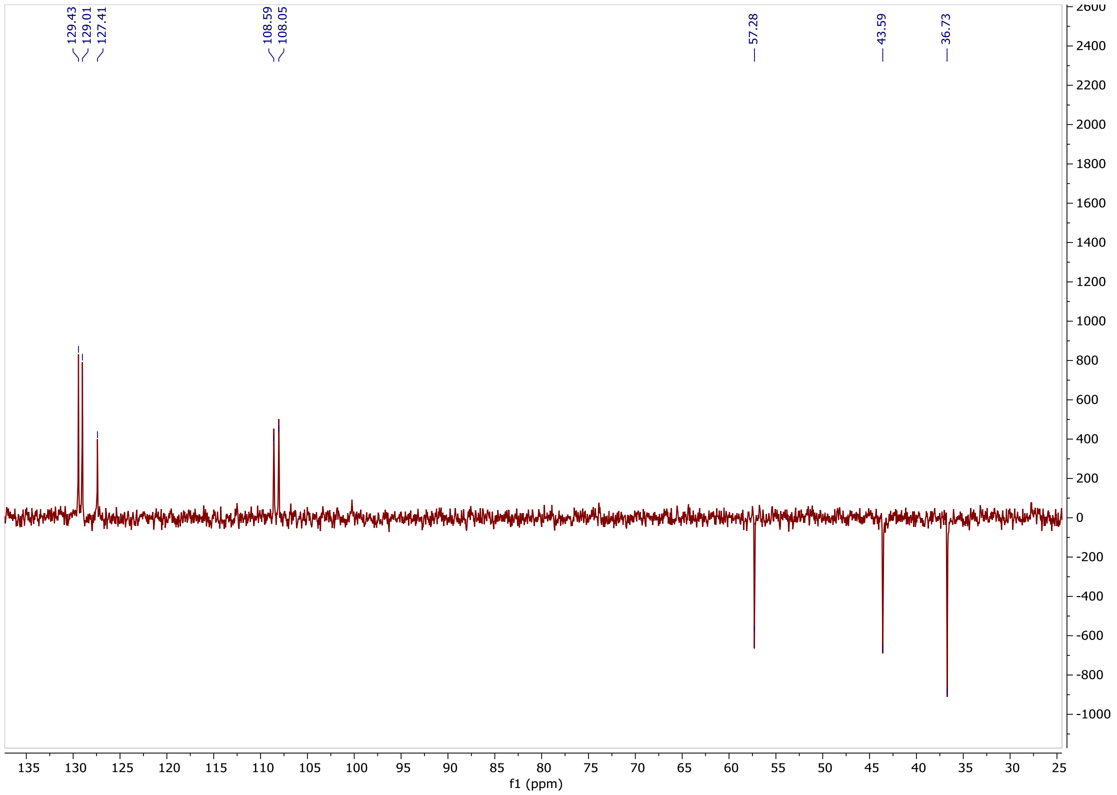

129.43
129.01
127.41
108.59
108.05
57.28
43.59
36.73
f1 (ppm)

N-[(5-(Hydroxymethyl)furan-2-yl)methyl]butyramide (7f)

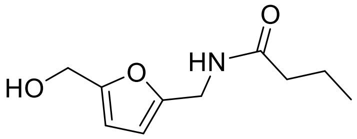

7f

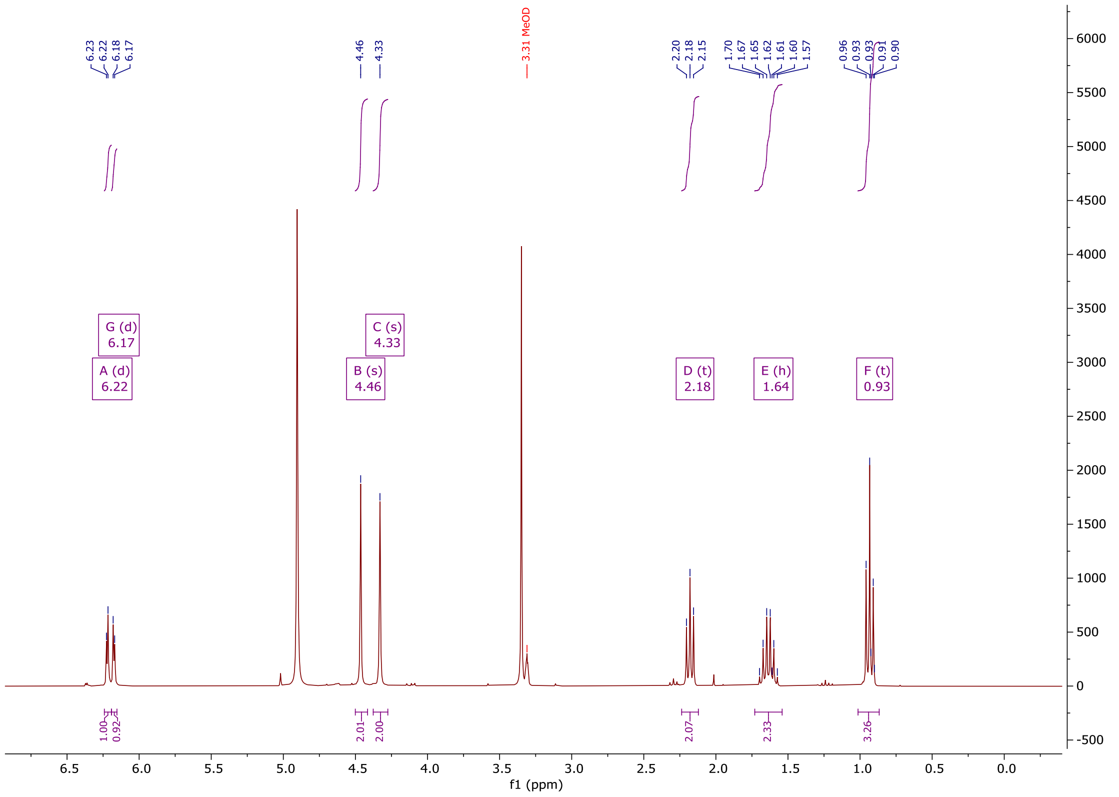

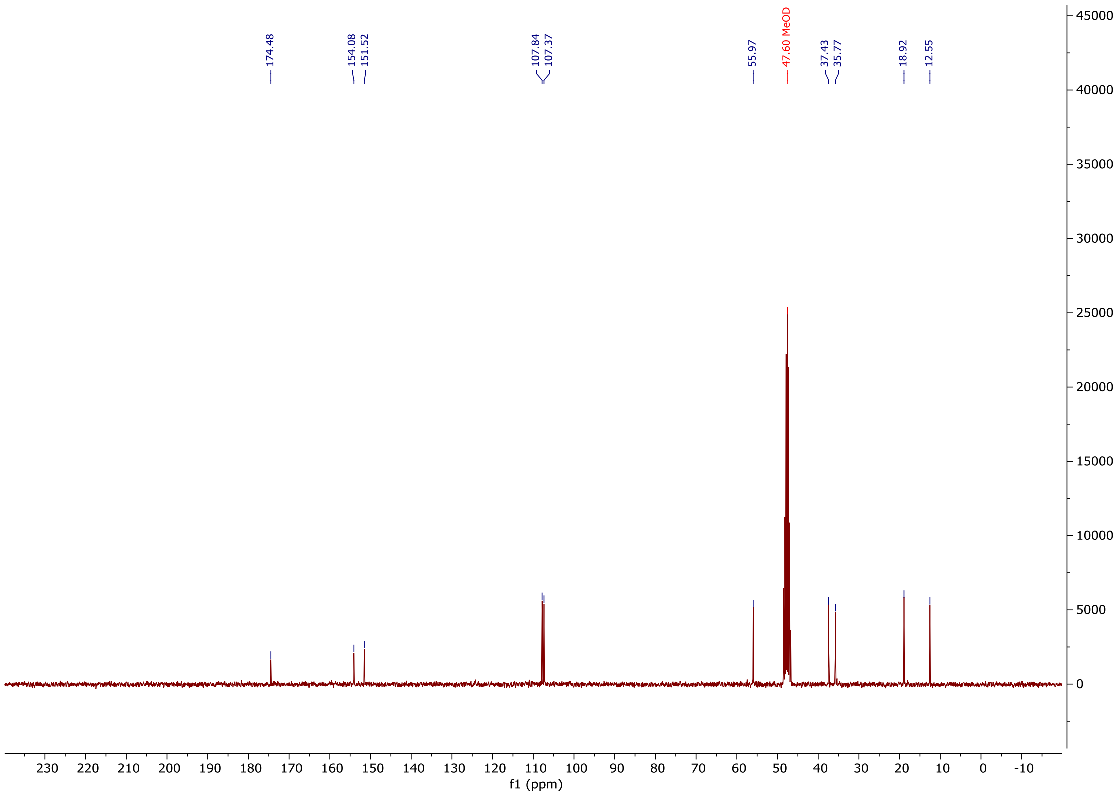

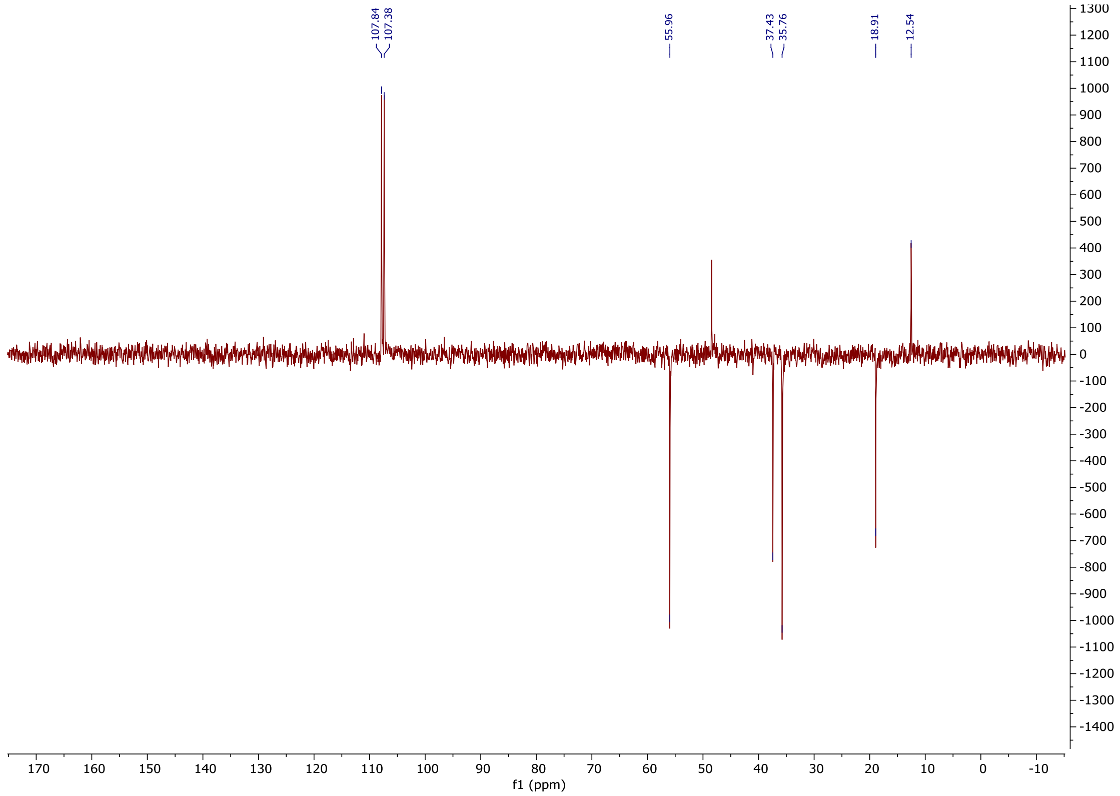

107.84
107.38
55.96
37.43
35.76
18.91
12.54
f1 (ppm)

N-[(5-(Hydroxymethyl)furan-2-yl)methyl]hexanamide (7g)

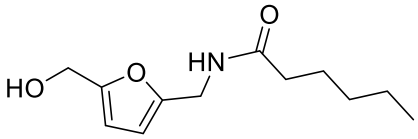

7g

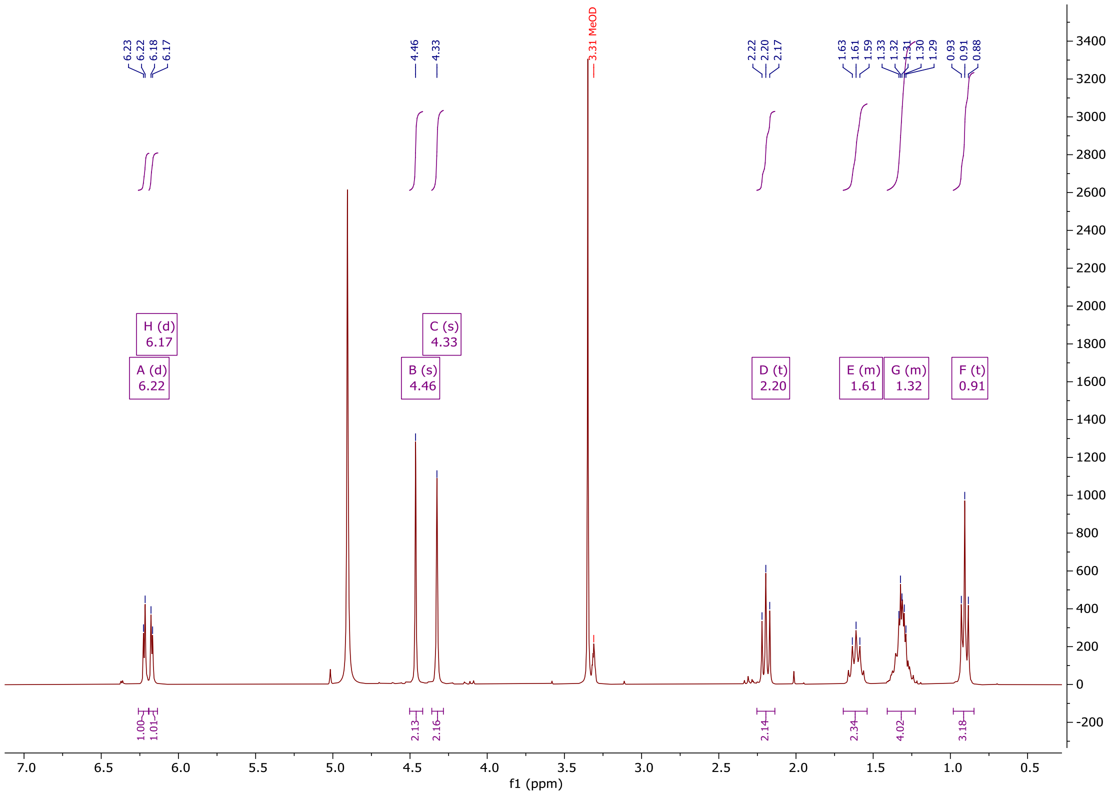

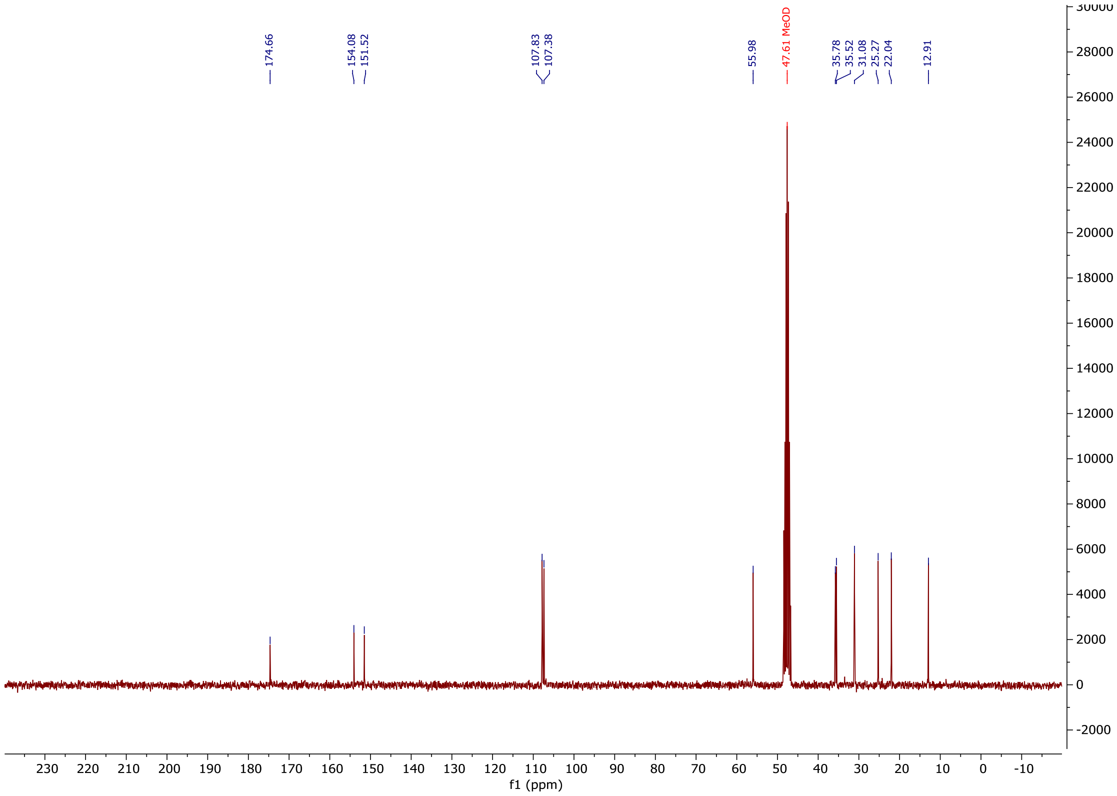
174.66
154.08
151.52
107.83
107.38
55.98
47.61 MeOD
35.78
35.52
31.08
25.27
22.04
12.91
f1 (ppm)

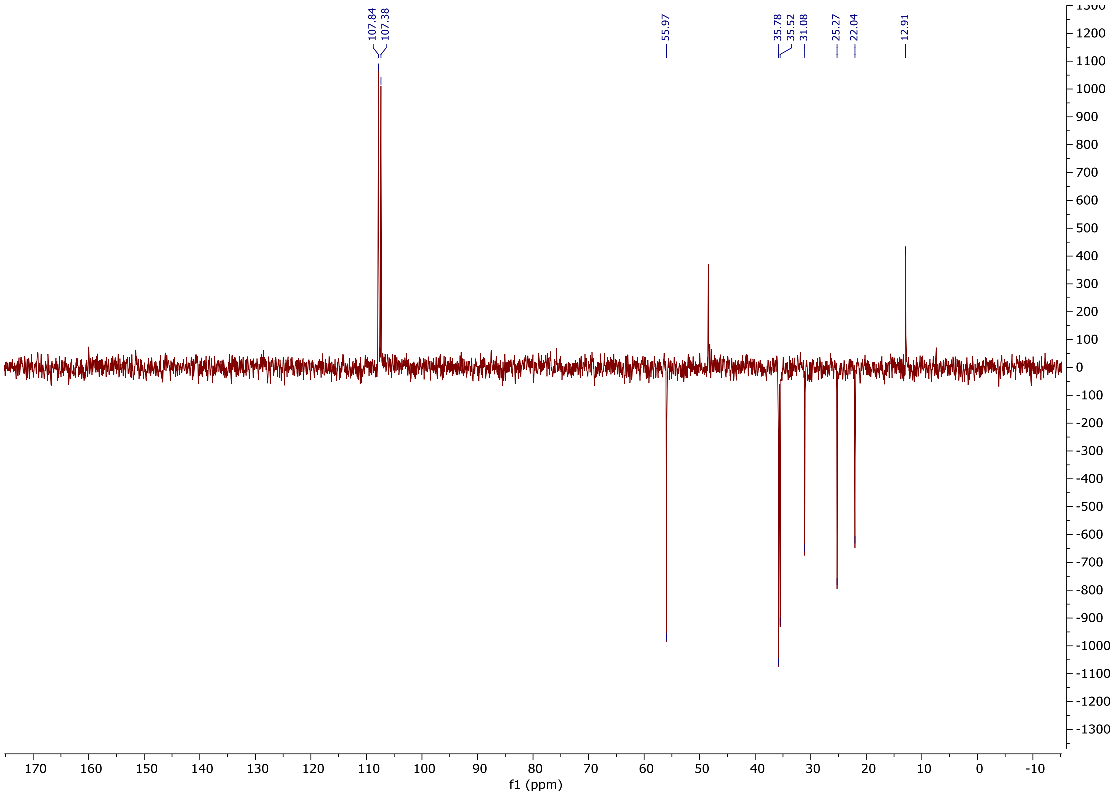
107.84
107.38
55.97
35.78
35.52
31.08
25.27
22.04
12.91
f1 (ppm)

N-[(5-(Hydroxymethyl)furan-2-yl)methyl]decanamide (7h)

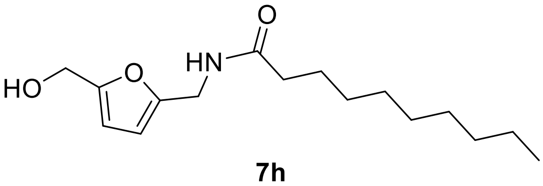

7h

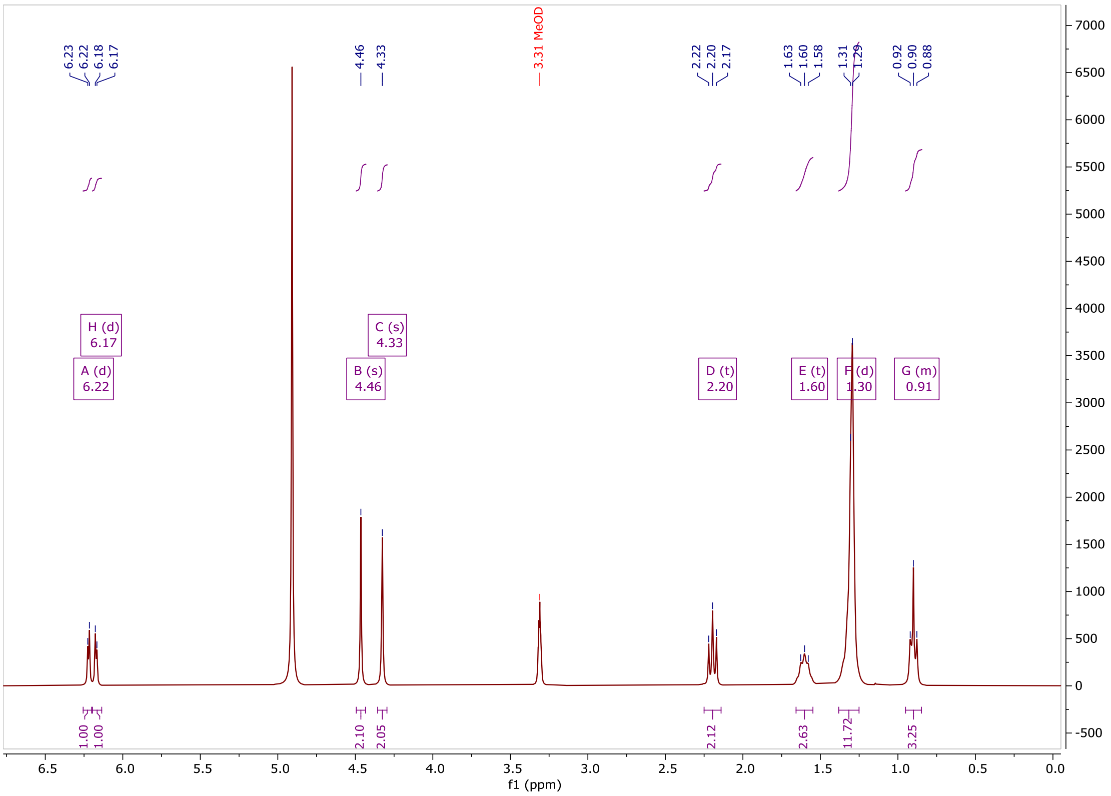

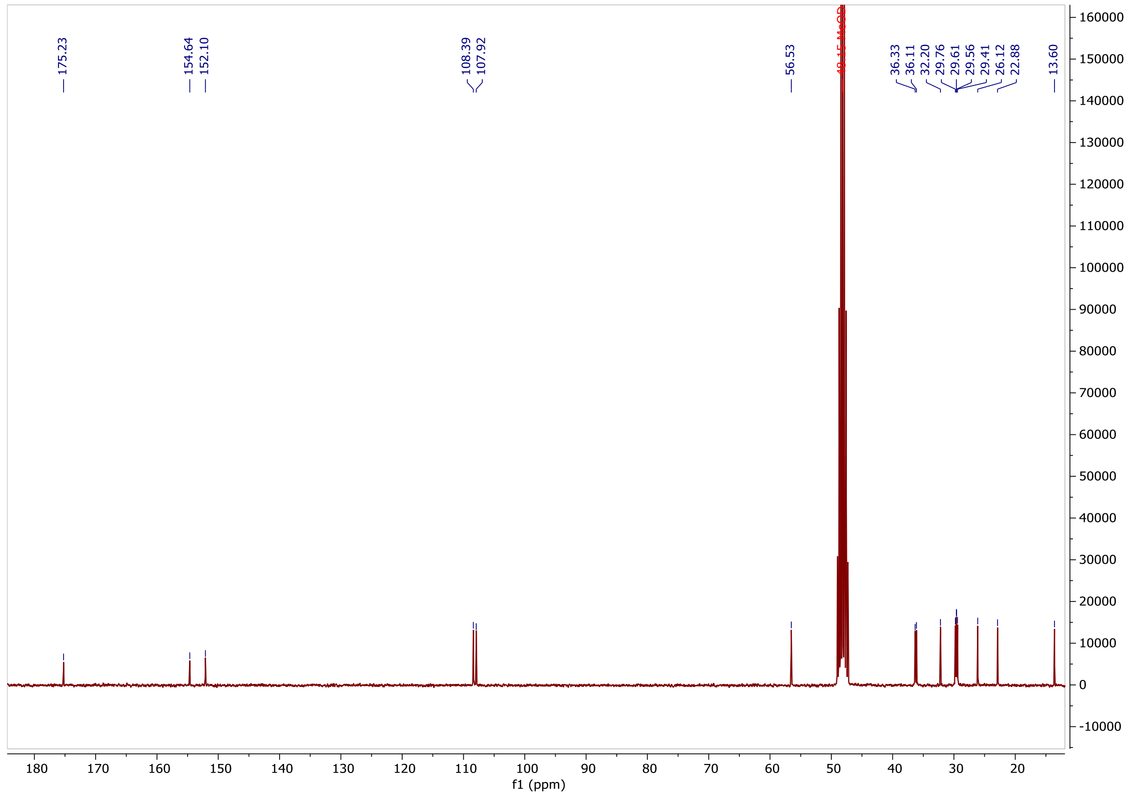
175.23
154.64
152.10
108.39
107.92
56.53
49.15 MeOD
36.33
36.11
32.20
29.76
29.61
29.56
29.41
26.12
22.88
13.60
f1 (ppm)

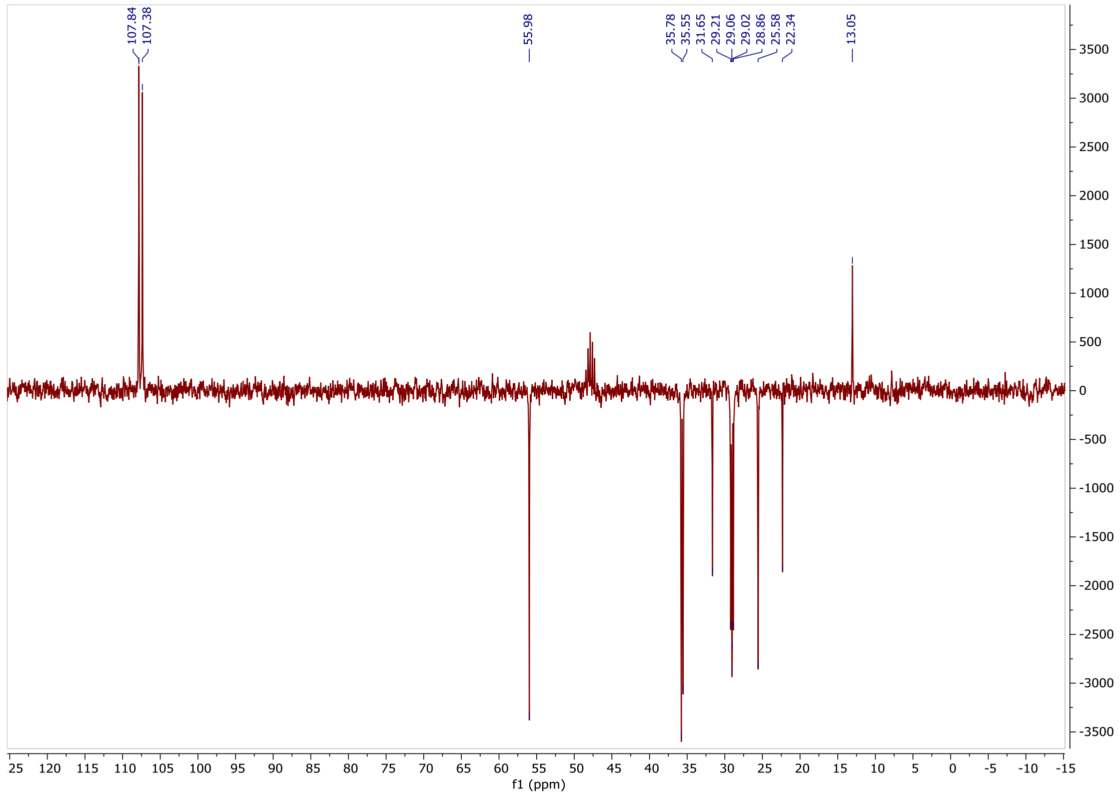
107.84
107.38
55.98
35.78
35.55
31.65
29.21
29.06
29.02
28.86
25.58
22.34
13.05
f1 (ppm)

N-[(5-(Hydroxymethyl)furan-2-yl)methyl]dodecanamide (7i)

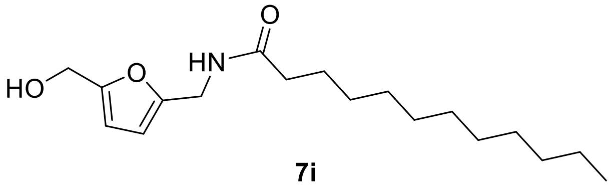

7i

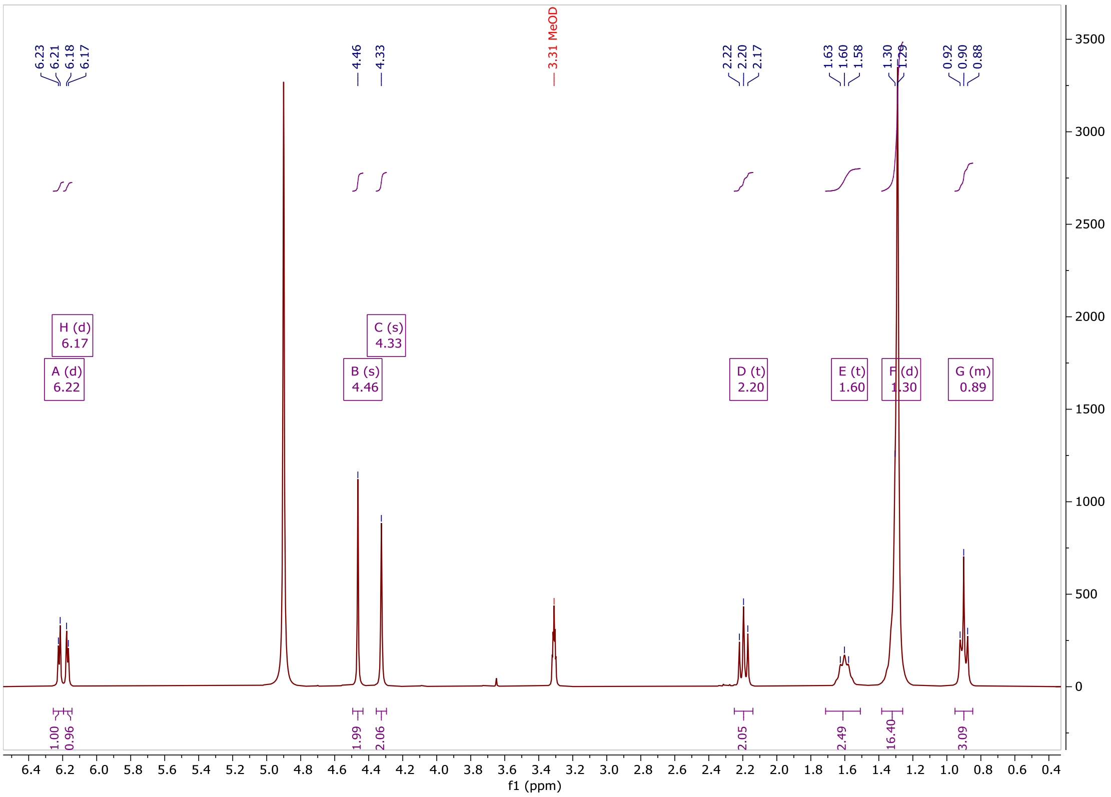

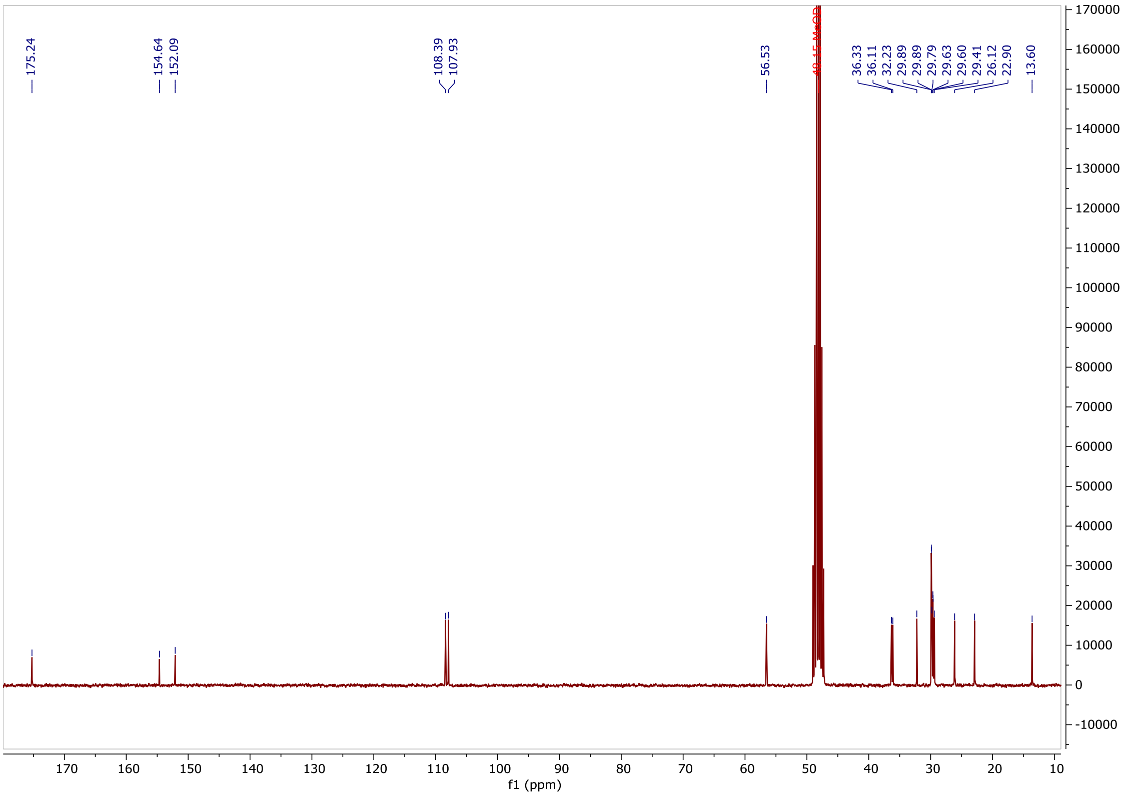
175.24
154.64
152.09
108.39
107.93
56.53
36.33
36.11
32.23
29.89
29.89
29.79
29.63
29.60
29.41
26.12
22.90
13.60
f1 (ppm)

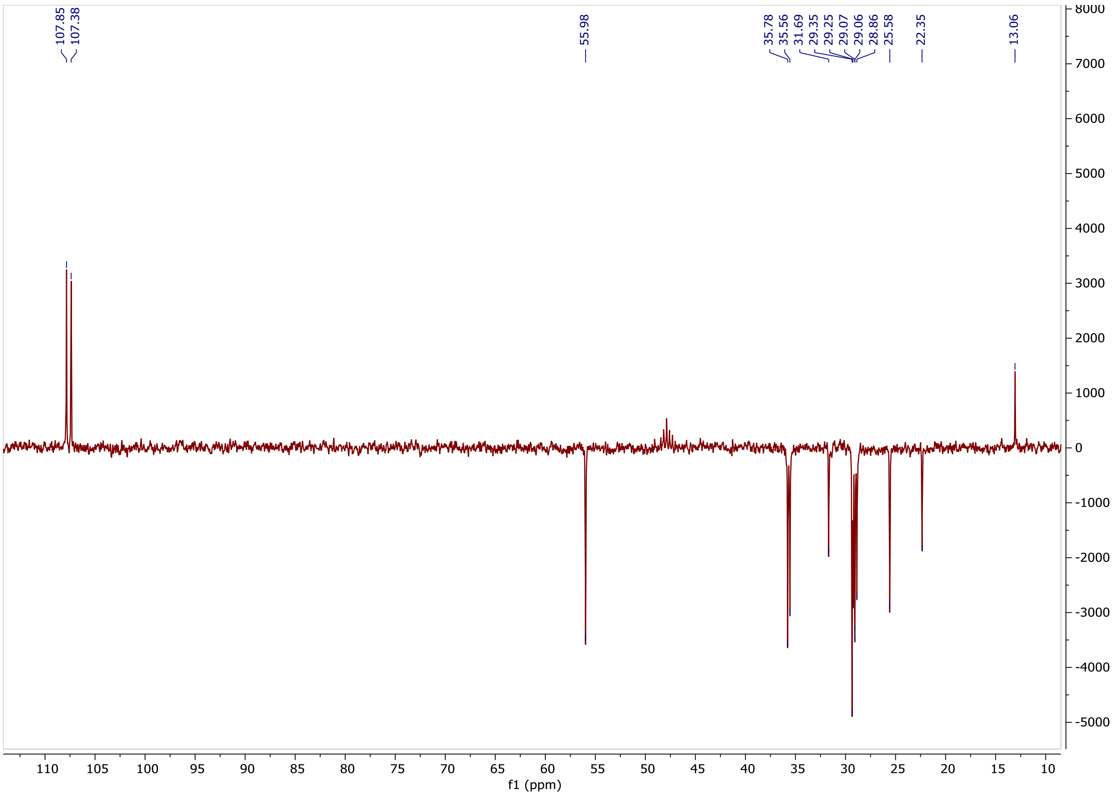
107.85
107.38
55.98
35.78
35.56
31.69
29.35
29.25
29.07
29.06
28.86
25.58
22.35
13.06
f1 (ppm)

{5-[(2-Methoxyacetamido)methyl]furan-2-yl}methyl acetate (10)

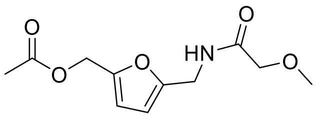

10

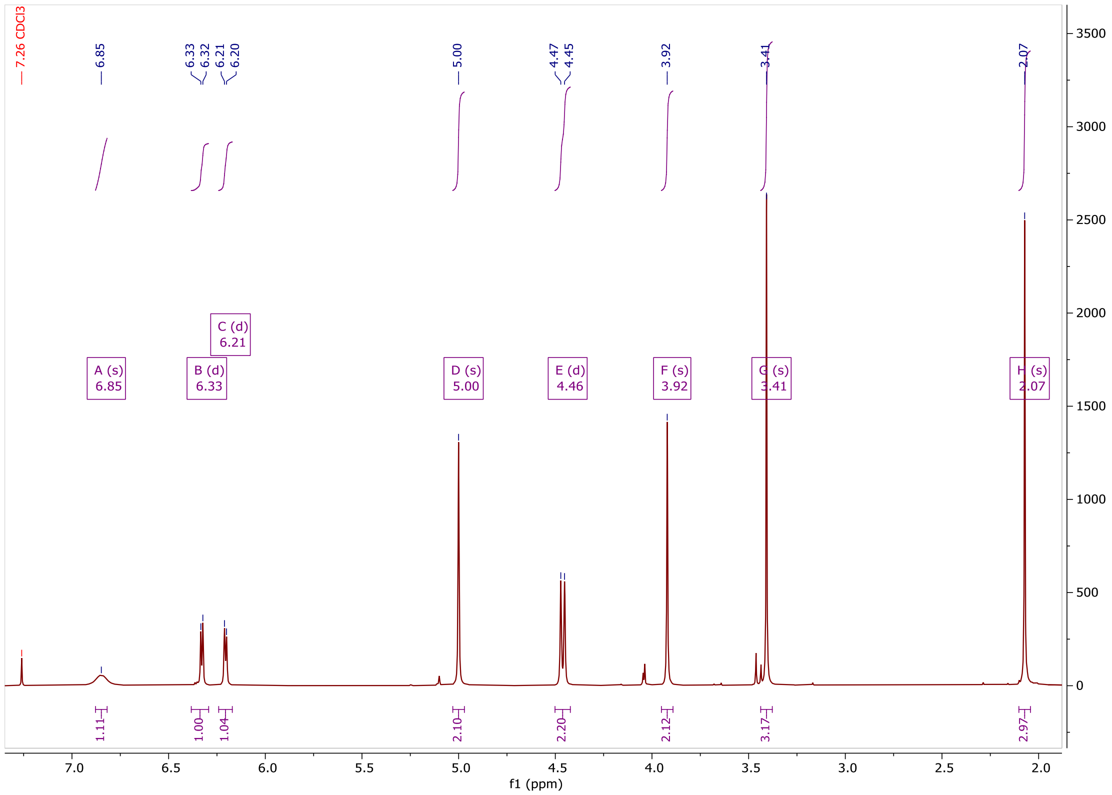

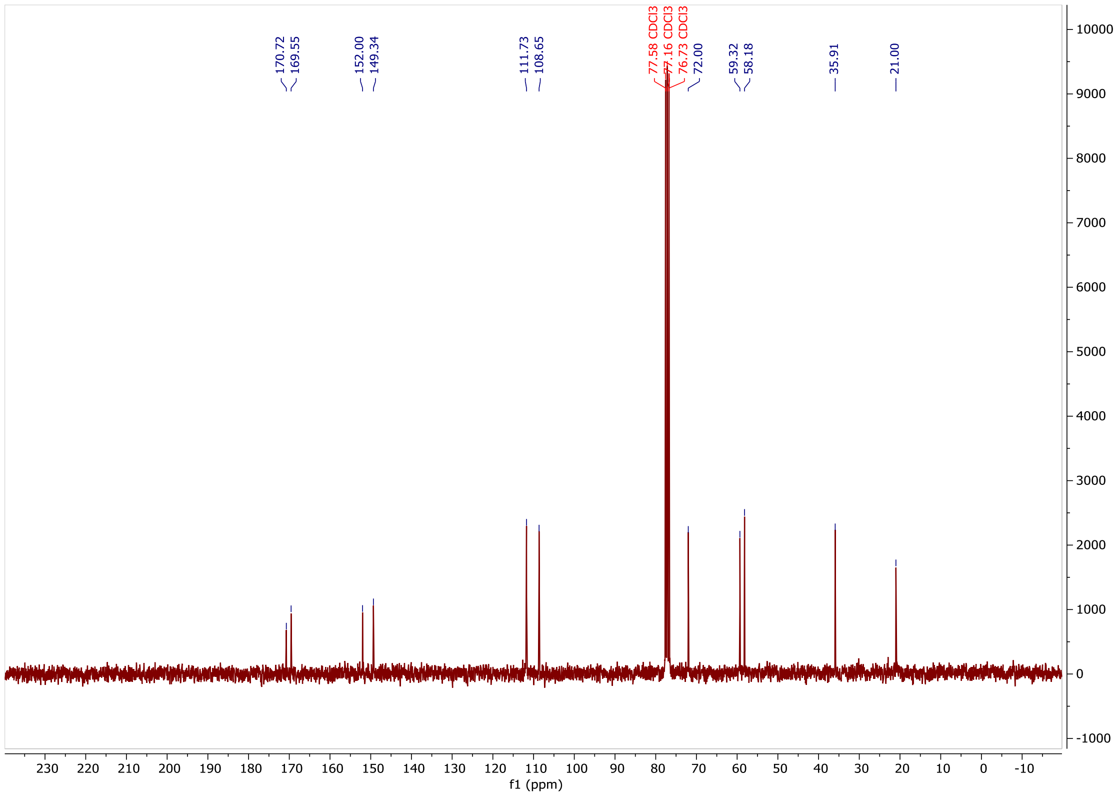
170.72
169.55
152.00
149.34
111.73
108.65
77.58 CDCl3
77.16 CDCl3
76.73 CDCl3
72.00
59.32
58.18
35.91
21.00
f1 (ppm)

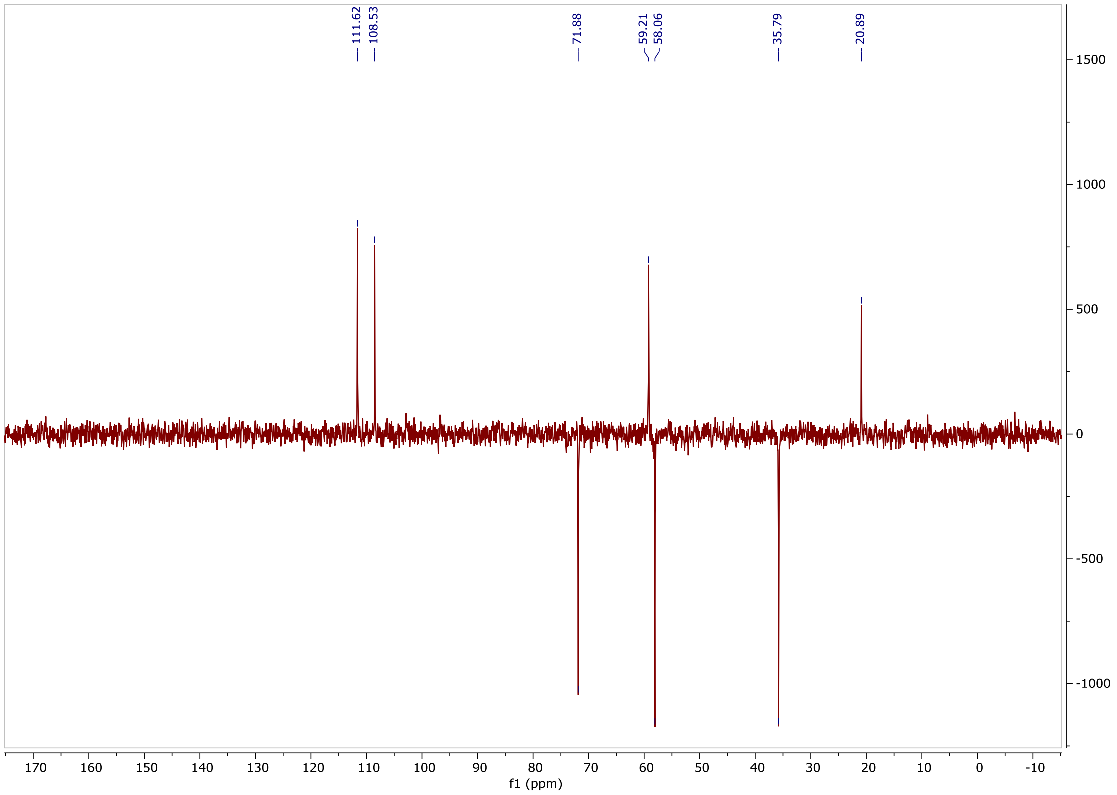
111.62
108.53
71.88
59.21
58.06
35.79
20.89
f1 (ppm)
